# Enhanced localized pressure-mediated non-viral gene delivery

**DOI:** 10.1007/s13346-025-01827-7

**Published:** 2025-03-12

**Authors:** James E. Dixon, Vanessa Wellington, Alaa Elnima, Amelie Savers, Lia A. Blokpoel Ferreras, Aveen R. Jalal, Hoda M. Eltaher

**Affiliations:** 1 Regenerative Medicine & Cellular Therapies Division, School of Pharmacy, The University of Nottingham Biodiscovery Institute (BDI), University of Nottingham, Nottingham, NG7 2RD UK; 2 NIHR Nottingham Biomedical Research Centre, University of Nottingham, Nottingham, NG7 2RD UK

**Keywords:** GAG-binding enhanced transduction (GET), Negative pressure wound therapy, Gene transfer, Transfection, Skin

## Abstract

Topically applied therapies must not only be effective at the molecular level but also efficiently access the target site which can be on milli/centimetre-scales. This bottleneck is particularly inhibitory for peptide and nucleic acid macromolecule drug delivery strategies, especially when aiming to target wounded, infected, and poorly perfused tissues of significant volume and geometry. Methods to drive fluid-flow or to enhance physical distribution of such formulations after local administration in accessible tissues (skin, eye, intestine) would be transformative in realizing the potential of such therapeutics. We previously developed a technology termed Glycosaminoglycan (GAG)-binding enhanced transduction (GET) to efficiently deliver a variety of cargoes intracellularly, using GAG-binding peptides and cell penetrating peptides (CPPs) in the form of nanoparticles. Herein, we demonstrate that the most simplistic GET formulation is relatively poor in diffusing into tissue matrix (tested in collagen scaffolds). Changing nanoparticle physicochemical properties can enhance penetration, however the use of a pressure differential, generating fluid-flow significantly enhances effective gene delivery over milli/centimetre scales. We adapted clinically used pressure systems to administer both negative (Negative pressure (NP) wound therapy; NPWT) and positive pressures (PP; Insufflator). Pressure differences generated enhanced distribution, and we were able to show for the first-time localized gene transfer in vitro in cell scaffolds and enhanced transfection of ex vivo skin explants. The ability to simply control intra-tissue localization of gene delivery on milli/centimetre scales using pressure application will facilitate new drug delivery strategies for accessible tissues. Importantly site-specific enhancement of penetration and activity of novel nanotechnologies and gene therapeutics could be transformative for future regenerative medicine strategies.

**Graphical Abstract:**

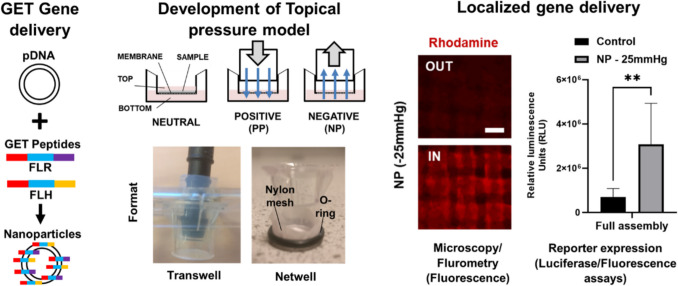

**Supplementary Information:**

The online version contains supplementary material available at 10.1007/s13346-025-01827-7.

## Introduction

Peptides and nucleic acid macromolecular drugs are highly specific, potent therapies that show great promise as novel systems for the treatment of many disorders [1]. Although they can offer many advantages compared with small molecule drugs such as high potency, low non-specific activity and toxicity [2], their clinical use has been inhibited. Specifically, nucleic acids like DNA or RNA are subjected to rapid physical and chemical degradation, short in vivo circulation half-life and biodistribution, and a lack of an efficient, safe, and specific delivery. In addition, clearance by the reticuloendothelial system, risk of immunogenicity, solubility challenges, and failure to permeate tissue bulk and cell membranes further reduce their therapeutic efficacy [3]. Thus, to achieve a high therapeutic efficacy of genetic strategies appropriate delivery platforms to enhance tissue distribution are vital.

Various methodologies have been developed to deliver therapeutic proteins and nucleic acids intracellularly [4–8]. Cell penetrating peptides (CPPs) can be tethered to therapeutics [9] and these trigger endocytosis-mediated uptake when they interact with the cell membrane [4]. Even though CPPs significantly increase cellular uptake, their activity requires them to be in vast extracellular excess (at micro-molar scales) to drive endocytosis. We have described the Glycosaminoglycan (GAG)-binding enhanced transduction (GET) system [10] that exploits membrane-docking peptides which bind heparan sulphate glycosaminoglycans (GAGs), combined with a CPP. Functional quantities of many cargos were successfully delivered either in media [10–12] or from scaffolds [13], biomaterials [11, 14] and hydrogels [15, 16] to cells. Importantly nanoparticles formed from GET peptides complexed with nucleic acids, such as plasmid (p)DNA have high transfection efficiency in vitro or in vivo [17–20]. Furthermore GET complexes can be delivered biolistically as a vaccine strategy [21] and can be applied to monogenic disease correction (such as for the lung [17]), and for regenerative medicine strategies [16, 18, 22–27] and drug delivery [28, 29]. Presently our most efficacious GET peptide is termed FLR (FGF2B-LK15-8R) that can be combined with an endosomal-escaping variant termed FLH (FGF2B-LK15-10H) [20, 30]. This combination can overcome some of the efficacy loss in difficult-to-transfect target cells. Additionally, to improve in vivo efficacy we have generated PEGylated versions for effective lung gene therapy applications [17] by having lower positive charge, reduced extracellular trapping, enhanced diffusion, as well as retaining high transfection efficiency.

In vivo, therapeutic macromolecules can be delivered by subcutaneous and intramuscular administration, intravenous infusion or topically [31–33]. However, as most macromolecules possess short biological half-lives in bodily fluids, this means that frequent regimens at high dosages are required for sustained effect [2]. Moreover, systemic delivery by nature does not allow targeted or localized delivery, meaning significant amounts of the administered therapy is miss-targeted [1, 34].

In order to overcome these limitations, biodegradable polymers and gel-based release systems have been used to allow controlled release of therapeutics locally at the disease site [35]. These controlled release approaches can be powerful as they extend the duration of activity, overcoming the need for frequent administration, and also implantation at the therapeutic site allows localized delivery to the tissue of interest [36–39]. We have recently described the use of GET gene delivery from temperature-sensitive hydrogel patches/bandages (employing Pluronic F-127 and Methylcellulose hydrogel) [16]. Transfection was effective with direct contact with cells, but only if the diffusion characteristics of GET nanoparticles in the hydrogels were improved, and if the formulations were used at very high concentrations. Even with these important demonstrations, the superficial localization of topical therapies means that, to be effective, they need to penetrate sometimes tens of millimetres to have full target-site effect. There is therefore a disconnect between the level of penetration needed to permeate through a large volume of target tissue and what can be effectively distributed either by topical contact or indeed invasive methods such as hypodermic injection for effective therapy tissue biodistribution.

Pressure-mediated enhancement of distribution of drugs and gene therapies, even as naked DNA, has been demonstrated for over a decade [40, 41]. This can be via systemic delivery in animal models followed by vacuum-suction applied to the tissue-of-interest (demonstrated for kidney and heart [42]) or via high-volume, high-concentration intravenous injections causing a transient increase in circulation pressure and enhanced permeabilization and delivery to the animal as a whole (with the vast majority targeting the liver) [43]. Invasive application of suction to tissues- or organs, and high-volume systemic injections are not clinically desirable approaches and more superficial demonstrations of effective targeting to wounds (such as for skin) are only starting to be demonstrated [40]. Local administration is an attractive route for targeted therapies, this is especially the case for percutaneous approaches as this does not cause pain, avoids hepatic first-pass metabolism and enhances patient compliance in long-term therapy [44]. Pressure systems are intensively applied to wounds via Negative-pressure wound therapy (NPWT) technologies [45] and also cupping technologies are being considered to administer negative pressure (NP) to enhance delivery [46, 47]. NPWT approaches have been extensively used in both acute (trauma, blast) injuries as well as transformative applications in chronic wounds, necrotic and poorly perfused tissue of the elderly and those with diabetes [48, 49]. If pressure could be used in a safe and deployable manner to improve the distribution of therapeutics to tissues, then efficacy of state-of-the-art nucleic acid technologies could be enhanced to be more clinically effective.

Here, in this study, we hypothesized that using the GET peptide delivery system along with the application of differentials of either positive (PP)- or negative (NP)- pressure environments could drive more effective localized therapeutic intracellular delivery of genes. We have already shown that atmospheric pressure changes per se do have some positive effect on transfection efficacy [30]. To provide exemplars of our concept, we examined how manipulating fluid-flow across samples could direct delivery and transfection in engineered tissues of cells and collagen scaffolds. GET peptide (FLR, FLH) and GET-plasmid (p)DNA nanoparticles were shown to poorly transduce and their delivery inhibited for thicker engineered tissues compared to enhanced activity seen in monolayer experiments. Some of this inhibition could be overcome by modifying the formulation by PEGylation (as previously [17]) or minimising diffusion distance needed (using thinner tissues). However, this did not achieve the millimetre diffusion we were aiming to demonstrate. By applying PP, or with more technical simplicity and translatability, NP, to engineered tissues we could achieve exceptionally high-levels of delivery and transfection, even in very diluted formulations by promoting fluid-flow through samples.

By applying localized vacuum from clinically deployed NPWT systems, we demonstrate that GET-delivery can be achieved in a zonal fashion within engineered tissue. Finally, the system was applied to porcine skin explants, with this ex vivo model demonstrating enhanced transfection with NP application. Our demonstration of successful and efficient gene transfer from pressure-mediated non-viral nanotechnology will open-up new avenues in regenerative therapies and drug delivery for superficial accessible tissue-targets.

## Materials and methods

### Materials

All materials were purchased from Sigma Aldrich (UK) unless stated. Dulbecco’s phosphate-buffered saline (DPBS) was provided by ThermoFisher. Pipework and connectors were obtained from Silex Silicones Ltd, UK.

### Cell culture

NIH3t3 fibroblast cells were cultured in Dulbecco’s modified Eagles media (DMEM; Sigma), supplemented with 10% (v/v) Foetal Bovine Serum (FBS, Sigma), 4.5 g/L D-Glucose, 2 mM L-glutamine and 100units/ml penicillin and 100units/ml streptomycin (Invitrogen). This media was defined as growth media (GM), the same media without FBS was defined as serum-free media (SFM) and cells cultured at 37 °C and humidified 5% CO_2_ as described previously [10].

### Collagen hydrogels and pig skin explants

Rat tail type-I collagen was purchased from Corning (High Concentration, 100 mg; Cat. No. 354249) and used according to manufacturer’s instructions (neutralized and buffered with PBS and NaOH). For transfections in 96-well plates, 12-well transwells or home-made netwells, and 50 ml tube cell strainers, we employed 20 µl, 500 µl and 200 µl volumes of collagen, respectively. Complete collagen formulations (with cells) were transferred to wells or membranes using wide-bore pipette tips and allowed to gel at 37^⁰^C for 30 min before addition of media and transfection. Freshly culled pig skin was obtained from a food grade abattoir. Skin sheets were cleaned with sterile water then 5% (w/v) Hibitane surgical scrub (Chlorhexidine) for 5 min. Hair and fat were removed using razor blades/scalpels, then washed with 70% IMS for 2 s, and several times with large volumes of PBS containing 2X Antibiotic/Antimycotic. Skin was then placed in at 37 °C and humidified 5% CO_2_ in fresh PBS containing 2X Antibiotic/Antimycotic for 1 h. Biopsy punches (8 mm) were generated from the processed skin sheet and placed into membrane-wells. The wells were plugged with 4% agarose in PBS to allow application of pressure across the sample.

### Cell metabolism and viability

Cell viability from monolayers and collagen hydrogels were assayed for cell metabolism using PrestoBlue™ (ThermoFisher, Cat no: A13262) as described previously [50, 51]. We employed 50 µl, 500 µl and 2 ml volumes for assays of 96-well plates, 12-well transwells/custom netwells, and 50 ml tube cell strainers, respectively. Time of incubation was varied with appropriate controls to allow significant colour changes before fluorometry in black 96 well plates (50 µl/sample). Cell suspensions were assayed for viability using trypan-blue exclusion and hemocytometer assessment. LIVE/DEAD™ (ThermoFisher, Cat no: L3224) was used following manufacturer’s instructions with modifications detailed previously [52].

### GET Peptides and pDNA preparation

Peptides were synthesized as previously described with or without a Rhodamine (TAMRA) fluorophore [12]. For luciferase assays, reporter plasmid (pDNA) expressing *gaussia luciferase (gluc)* was acquired from New England Biolabs (pCMV-gluc2 termed pGluc) [18]. For fluorescently tracing pDNA and GET-pDNA pGluc was labelled with Rhodamine (TAMRA) using the *Label* IT® Nucleic Acid labelling kit (TM-Rhodamine) following the manufacturers’ instructions (Mirus Ltd). 5 μL of 10X Labelling Buffer A in 35 μL of nuclease-free water was mixed with 5 μL of 1 mg/mL pDNA and 5 μL of a Label IT TAMRA Rhodamine Reagent. The mix was incubated for 1 h at 37 °C. Labelled pDNA was purified using a G50 MicroSpin purification column and was then stored protected from light at − 20 °C. For fluorescent reporter assays, *enhanced green fluorescent protein (eGFP)* expressing pDNA was acquired from Takada, Japan (pEGFP-C1 termed peGFP). Both plasmids are driven by an enhanced cytomegalovirus (CMV) promoter. The plasmids were transformed in DH5α competent *E.coli* cells and purified by endo-free Maxi-prep kits (Qiagen, UK) as previously [17].

### GET-pDNA complexation and transfection

Our conventional GET-plasmid (p)DNA complexation methodology was modified and scaled to the volumes required [17, 30]. Typically for 96-well transfections we used high cell densities (2.5 × 10^4^ NIH3t3 cells) and delivered 0.125 µg pDNA (in 6.25 µl SFM) complexed with 0.1 µl FLR and 0.125 µl FLH (1 mM) (in 6.25 µl SFM) creating a 12.5 µl transfection volume. These were then combined, mixed, complexed for 15 min at room temperature, and then added to samples (containing 50 µl media). For collagen and skin transfections requiring larger pDNA dosages, the complexation was scaled within these pDNA:FLR:FLH charge ratios (Charge ratio 8). The maximum pDNA concentration used for complexation was 4 µg pDNA in 25 µl transfection volume to enable faithful GET-pDNA complex generation. For higher dosage experiments, larger complexation volumes were employed.

### Fluorometric analyses of delivery

Fluorescence of Rhodamine, Rhodamine (TAMRA) TM-labelled FLR peptide and Rhodamine (TAMRA) TM-labelled pDNA (pGluc) to generate labelled GET-pDNA complexes were used to measure fluid-flow and diffusion through collagen with pressure. TRITC-labelled Dextran (5.5 kDa) was also employed to assess diffusion. Collagen cryosectioning prior to fluorometry was conducted as previously described [15].

### Application of pressure differentials to wells

We employed RENASYS NPWT and Insufflator 500 (Smith & Nephew) systems to achieve regulated NP and PP, respectively. We employed silicon-pipe connectors and a manifold to allow multiple samples to be tested simultaneously. The systems were installed into a conventional culture incubator so that NP and PP differentials could be applied in controlled conditions. To achieve pressure differentials across cultured samples we used 12-well transwell inserts (0.4 µm pore polyester membranes; Corning) or inserts modified to replace the membranes with larger pore nylon meshes (Plastok Associates Ltd, UK). To generate transwells, termed ‘netwells’ here, we removed the original membrane by scalpel and replaced the membrane with 1 to 75 µm pore mesh (drawing the mesh tort with placement of a securing silicon O-ring), these were washed in 70% IMS and UV sterilized before casting of collagen gels onto the mesh. Netwells were placed in modified 12-well plates (with 12 mm holes drilled centrally of the well in the lids). An 8 mm inner diameter (2 mm thick) silicon pipe was secured, through the holes, which plumbed directly into the inner of the transwell. The interface between the collagen gel was filled with foam sponge (sterilized, 12 mm diameter, 6 mm thickness; Sherlock Foams, UK) to promote fluid flow. For NP this was within the extraction pipe fitted to the transwell and for PP this was between the under surface of the transwell and the well in which it was placed. For collagen samples it was important that the full surface of the netwell was sealed with crosslinked gel (500 µl/net well), if this were not the case the fluid-flow would bypass the sample. Collagen gels and pig skin explants (sealed with agarose) in netwells were covered by 500 μL GM after set-up and used immediately for transfection experiments. Modified 12-well plates were sufficient to house short-term PP or NP delivery experiments (maximum 2 ml), however large volumes of delivery media were required to apply either PP or NP for extensive periods of time (> 1 h). The well plate system was substituted by placing the netwell system into a large volume of media in a falcon tube for NP or by loading the large delivery media volume into the insufflator system above the samples.

### Luciferase reporter assays

Secreted luciferase reporter levels were measured at different times post-transfection by plate-reader luminometer (TECAN Infinite 200 PRO) and compared with controls as previously described [17, 18].

### Fluorescence microscopy and flow cytometry

eGFP expression and Rhodamine (TAMRA) labelled-pDNA fluorescence in cells was assessed by fluorescence microscopy and flow cytometry. Transfected cells as monolayers or collagen gels were washed twice with PBS and imaged by confocal microscopy (Leica TCS SPE) using a green laser for Rhodamine labelled pDNA and blue laser for eGFP. For flow cytometry, monolayer cultured cells were trypsinized with trypsin/EDTA (0.25% (w/v) trypsin/ 2 mM EDTA) and fixed with 4% (w/v) paraformaldehyde (PFA). Collagen gel-loaded cells were recovered enzymatically (in 200 µl volumes) as previously described [10] and fixed in 4% w/v PFA for analysis. GET nanoparticle cellular internalization was quantified using a Beckman Astrios Cell Sorter and 590 nm laser (20,000 cells minimum, gated on untreated cells by forward/side scatter). eGFP reporter expression was quantified using a 488 nm laser. Mean fluorescence intensity (MFI) was used for statistical analysis. Scatter plot and histogram graphs were produced using Kaluza flow cytometry analysis software.

### Targeted transfection using pressure

To generate cell-laden Collagen hydrogels with targeted/untargeted regions for transfections (IN and OUT areas) the surface of cell strainers (40 µm pore-size, 20 mm diameter, 314.16mm^2^) was marked with a circle of 6 mm diameter (28.27mm^2^) centrally using a template and pencil, defining the IN area. Collagen hydrogels were then cast onto the mesh and set. To confirm targeting of fluid-flow to the IN-region, media in the lower well (2 ml) was blue dyed with E133 colouring (10 µM; Acid Blue 9/Erioglaucine disodium salt; Sigma, Cat no. 861146) to visually inspect the targeted area and to record the application accuracy. NP was applied to the upper collagen surface with a 6 mm applicator tubing attached to the RENASYS system and suction held the sample and device to allow application of NP and fluid-flow. After application, the cell-strainer was washed in PBS and imaged. For transfection, the IN and OUT regions (the remaining area outside the central 6 mm diameter ~ 285.5 mm^2^) were cut away with a scalpel and incubated for 24 h separately to express the reporter gene delivered (Gluc assessed by Luciferase assay, Rhodamine (TAMRA) TM-labelled pDNA (pGluc) assessed by microscopy).

### Statistical analysis

Statistical analysis and graphs were generated using GraphPad Prism software package. Unpaired t-test and One-way ANOVA were used to determine significant variances between two groups or more respectively. Two-way ANOVA was used for grouped data. One-way and two-way ANOVA were followed by Tukey test to determine significance between each mean in multiple comparison. The data represented as mean ± SD. Variances between means were considered statistically significant with p values < 0.05 (*), 0.01 (**), 0.002 (***), 0.001 (****). Experimental numbers were a minimum of three biological replicates in every experiment.

## Results

### Transfection of engineered tissues is inhibited by diffusion

**Fig. 1:**
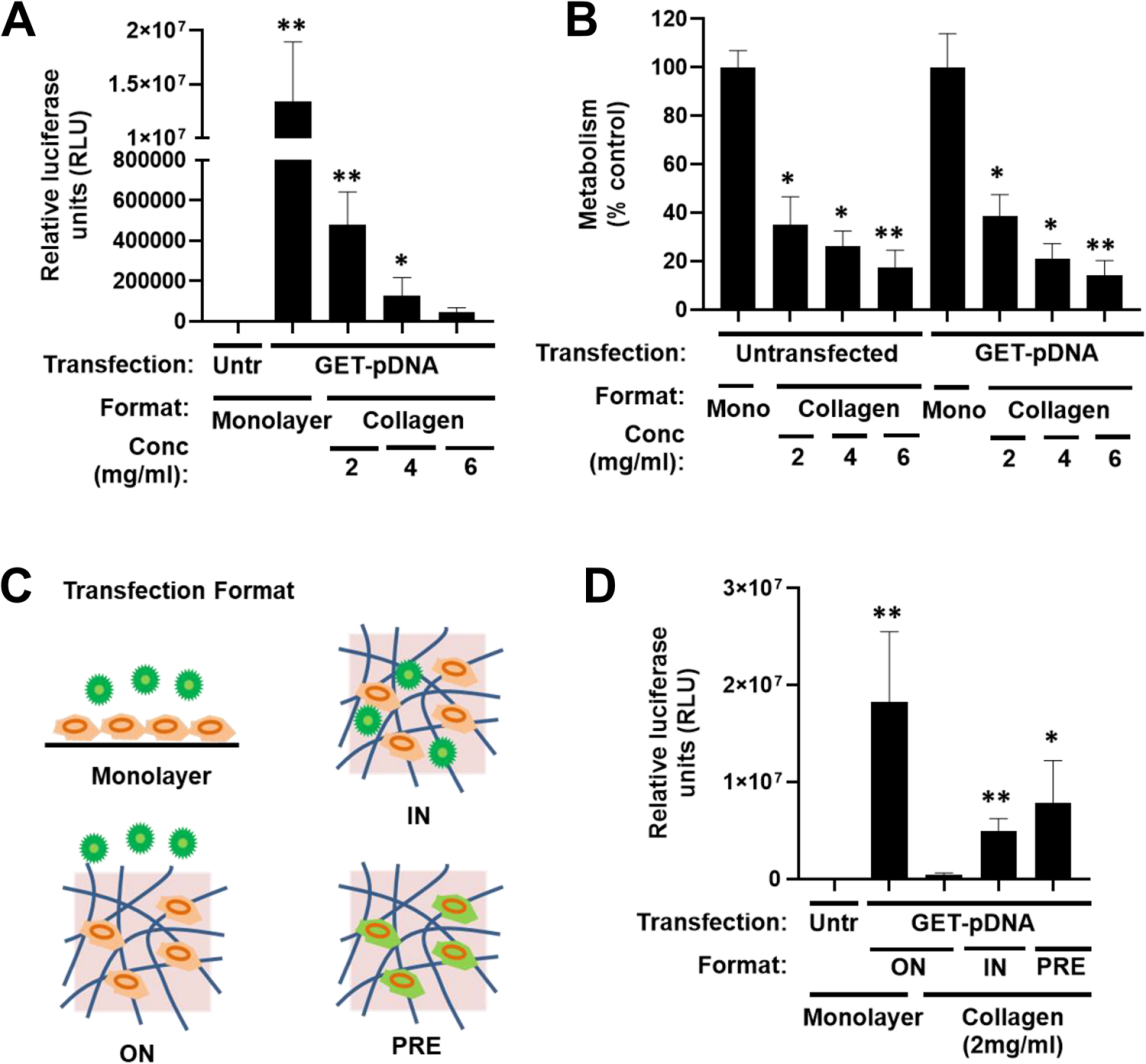
Poor penetration of complexes when transfecting collagen hydrogel-laden cells at atmospheric pressure (**A**) Luciferase assay of pGluc pDNA transfection in the ON format of NIH3t3 cells in monolayers or in collagen hydrogels (2–6 mg/ml) using FLR:FLH and pDNA (GET-pDNA) nanoparticles at 24 h using 1X dose (1 µg/ml). **B** Metabolic activity (PrestoBlue) of NIH3t3 cell monolayers or collagen cells untransfected or transfected as in A). **C** Schematic of the formats used to transfect NIH3t3s in monolayer or in collagen hydrogels. **D** Comparison Luciferase assay of pGluc pDNA transfection of NIH3t3 cells in monolayers or in the ON, IN or PRE-(transfected) formats in collagen hydrogels (2 mg/ml) using FLR:FLH at 24 h using 1X dose (1 µg/ml). Data was normalized to untransfected (Untr) monolayer as 100%. (*N* = 6, bars are S.D. ** *p* < 0.01, * *p* < 0.05)

We recently characterized the important variables for enhancing transfection from our GET system [30]. Very low dose pDNA transfections can be successful by employing an optimized endosomal-escaping variant formulation (FLR:FLH; [20]), and having optimal microenvironmental conditions [30]. As our aim was to demonstrate effective transfection in 3D, and to confirm diffusion through extracellular matrix in a thick material, we assessed transfection variables using collagen gels as a model engineered tissue. Native human dermis is formed from densely packed thick collagen bundles with diameters ranging from 20 to 50 µm interwoven with loose collagen fibres [53] constituting 75% of the dry weight of the skin [54]. From these perspectives 3D collagen gels have been extensively utilized as primary ECM tissue models [53, 55]. Collagen hydrogels are not as complex as native ECM but they are the most essential component of the extracellular skin matrix [55] that provide the density and multi-micron porosity needed to assess penetration of nanocomplexes and transfection in the presence of cells. Collagen gel pore-size has been extensively characterized previously having ~ 6–11 µm pores from collagen crosslinked in a similar methodology (1.5–3 mg/ml). Pore size decreases with increasing collagen concentration [56]. NIH3t3 cells cast in ~ 200–300 µm thickness rat tail type-1 collagen hydrogels as the engineered tissue were transfected with GET-pDNA complexes at a variety of gel concentrations (Fig. 1). Collagen gels were viscous and set within 30 min, meaning that cells did not sediment and were evenly distributed throughout the gel volume*.* We found transfection efficiency was highly dependent on gel concentration; all stiffnesses/densities (2–6 mg/ml collagen) could achieve transfection; however this was significantly lower (inverse to collagen concentration) than monolayer cultures. We hypothesized this was due to penetration of the GET-pDNA complexes into the matrix (1.34 ± 0.55 × 10^7^ versus 4.78 ± 1.6 × 10^5^ RLU for monolayer and 2 mg/ml collagen, respectively) (Fig. 1A) and was not due to cytotoxicity (Fig. 1B). To confirm this, we used either pre-transfected cells loaded (Matrix control) or cells and GET-pDNA complexes loaded into collagen hydrogels (transfection control) (Fig. 1C, D and S1). Cells pre-transfected (PRE) or transfected by co-loading GET-pDNA complexes with cells during collagen hydrogel fabrication (IN) achieved significantly higher transfection than those exposed to GET-pDNA complexes post-gelation (ON) (7.9 ± 4.4 × 10^6^ and 5.0 ± 1.2 × 10^6^ vs 4.8 ± 1.6 × 10^5^ RLU for 2 mg/ml collagen, respectively) (Fig. 1B). We further confirmed penetration being an issue with fluorometry of sectioned gels delivering fluorescently labelled pDNA (TAMRA-pDNA), with the majority of both being confined to the first 100 µm of the gel (Figure S2). This confirmed that collagen itself and bulk volume was indeed capable of supporting transfection and Gluc secretion per se but was likely to be poorly transfected due to insufficient penetration of the GET-pDNA complexes through its thick geometry.

To confirm that this phenomenon was not specific to GET transfection we completed experiments with Lipofectamine, which is a conventional lipid-based transfection reagent (Figure S3). We showed that compared to GET, Lipofectamine 2000 transfection was much more profoundly inhibited in collagen gel transfections. It is known that this system aggregates readily and it is highly likely that these particles are prevented from diffusion into the gel and therefore transfection of laden cells.

Experiments were repeated with eGFP pDNA transfection, and for quantitative analyses the collagen scaffolds were digested with collagenase and resultant cells separately collected for flow cytometry quantifying eGFP expression. It was evident that vast majority of cells transfected were those exposed to media on the upper surface (Figure S4) or on the extremities of the gels (not shown). We next repeated experiments where thicker (low surface area-to-volume; ~ 500 µm thickness) and thinner (high surface area-to-volume; ~ 100 µm thickness) collagen gels were generated. Thinner gels transfected more efficiently (~ 5.1-fold increase) than thicker gels in agreement with a penetration-deficiency hypothesis (Figure S4). Although transfection efficiency was low, serial delivery improved this (40% positivity on day 3 post-transfection) with no significant effect on metabolic activity (estimating viability) (Figure S5).

To understand the diffusion issue more thoroughly we employed our PEGylated formulation which is proven to diffuse better through complex biological solutions (such as mucus) [17] than the conventional GET nanoparticle formulations (Figure S6). Furthermore, PEGylated formulations are resistant to aggregation is solutions such as blood or serum, meaning they perform better in in vivo applications. In vitro*,* PEGylated formulations (40% PEGylated FLR) transfect monolayer significantly lower than the parent peptide FLR [17] (~ 50- and tenfold lower for PEGylated, for monolayer and thin collagen, respectively). However, proportionately PEGylated formulations were less affected (than non-PEGylated formulations) by thickness (~ 2.1-fold higher in thinner than thicker hydrogels) suggesting that diffusion through the gel and interaction with cells was less inhibited for PEGylated formulations. As GET-pDNA formulations do transfect collagen-loaded cells albeit with inhibited efficiency, collagen does not directly inhibit transfection per se, but the thickness of material does.

We sort to determine if simply increasing GET-pDNA complex dose could improve the transfection of thicker hydrogels (Figure S7). We increased the standard pDNA dose (1 µg, termed 1X) up to tenfold higher (10X) and conducted monolayer, thin and thick hydrogel testing. Very high formulation concentrations were found to be cytotoxic for monolayer and thin gel cultures (Figure S7, data normalized to monolayer viability at different doses), however there was a marked improvement (~ 8.4-fold) in luciferase levels in the thick gel samples. We therefore argue that GET-pDNA complexes without PEGylation retain the function to transfect cells in the presence of collagen, however their diffusion to allow interaction and uptake by cells is poor; therefore their activity overall is inhibited as a transfection agent.

### Development of a cell culture system to apply pressure-differences across engineered tissues

**Fig. 2:**
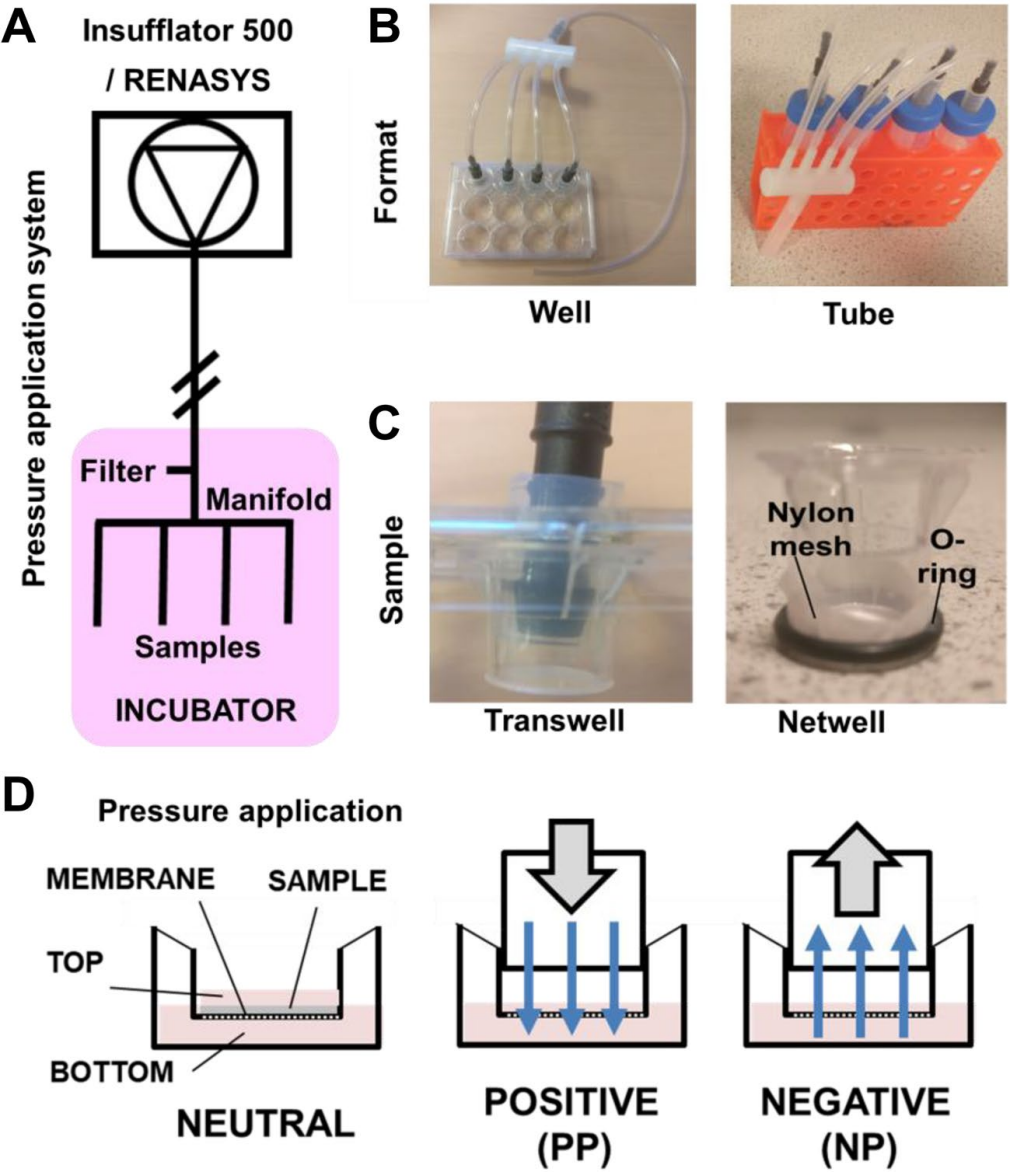
Pressure differential application tissue culture system using approved clinical devices. **A** Schematic of the pressure application system using clinical devices (Insufflator 500 for PP, RENASYS for NP; Smith & Nephew). These were attached external of incubators with silicon pipework into a conventional tissue culture incubator. A 0.2 µm filter was placed into the line as a leak to allow regulation of the PP or NP by the clinical devices. The line was then split by a 4-way manifold to allow application of pressure differential across 4 samples. **B** Two systems were developed to allow samples to be placed in wells and tubes, which allows smaller or larger volumes to be used for incubation across hydrogels or tissue. **C** Commercial transwell set-up with connection and sponge to maintain hydrogel/tissue position. Custom netwells were generated with nylon mesh to test the effect of different pore-sizes. **D** Schematic of sample position in the transwell/netwell system with pressure and liquid flow from top to bottom (Positive pressure, PP) and from bottom to top (Negative pressure, NP)

We hypothesized that if fluid-flow could be enhanced across the engineered tissue model then there would be the possibility of enhancing diffusion and therefore the effectiveness of GET-pDNA transfection in tissues. We adapted conventional well plate culture to test both NP and PP systems aimed to generate a pressure differential across tissue and promote fluid-flow (Fig. 2). We modified 12-well transwells to produce a culture adaptor facilitating application of NP or PP differentials to the top of the transwell while submerged in media in bottom well. For administration of NP (down to −200 mmHg) we employed a commercial clinically deployed NPWT system (RENASYS; Smith & Nephew) which could be directly connected to the transwell with silicon/PVC pipework (Fig. 2A). This system self-regulates and can apply the same pressures/differentials as that applied clinically. In the same fashion we employed an Insufflator (500 Insufflator; Smith & Nephew) usually used in laparoscopic surgery to apply PP (up to 30 mmHg) across samples. NPWT extracts air/liquid on NP administration and Insufflators when applying PP do so with pre-warmed gas, meaning that temperature differences during treatment would not confound the system during testing. Furthermore, both the NPWT and Insufflator systems used have pressure feedback sensors to control delivery of the condition real-time. Pressure adjustment was vital to maintain the stabilization of the system and corrected for some minimal leakage from the culture vessels/pipework (with an inline filter). Using silicon tubing which can connect transwells placed in both 12 well plates and sterile screw-top tubes (of any size, using 50 ml tubes) and a manifold (Fig. 2B), we were able to set up 8 samples (2 sets of 4 under different pressures) and 4 controls meaning that quadruplicate biological replicates could be set up under control and 2 conditions simultaneously. This was immensely beneficial allowing statistically significant data to be generated and many variables to be tested within a relative short experimental time. Initially we employed commercially available transwells in this system (Fig. 2C). It was possible to achieve stable pressure differentials across an empty 0.4 µm transwells and those with ~ 500 µm thickness collagen gels (2–6 mg/ml) with NP (down to −200 mmHg) and PP (up to + 30 mmHg) systems. The higher the pressure differential and lower the impedance (i.e., without a gel) the faster liquid would transfer from the atmospheric side (well or tube side) through the transwell pores (and gel) and into the space above the sample for NP, and vice-versa for PP. For NP, depending on volume media would travel into the manifold (if using 45 ml atmospheric volume in the set up). For higher flow using PP, large volumes were required so tubing length connected to the systems was extended to allow collection or delivery of individual samples with NP and PP, respectively. High NP differential emptied the well set up (which contained 1.5 ml) very rapidly (< 5 min) meaning that only low (25–50 mmHg) pressures were used with wells and higher pressures required the higher volumes of the tube set up. Also, higher NP had a higher tendency to displace and bypass collagen gels meaning that sterile pharmaceutical grade open-pore sponges were placed above the collagen samples holding them down onto the transwell surface. This sponge was pressed in place using the connecting silicon tubing meaning that the collagen gel was firmly held in place to avoid liquid bypass (but not compressed). The system is used in a cell-culture incubator meaning evaporation was not apparent if the differential volume did not run dry (due to removal of all the liquid by the pressure differential). The system was therefore maintained accurately at 37⁰C and 5% CO_2_ culture conditions by placing all the set-up inside an incubator (Fig. 2A, D).

### Transfer across a membrane using passive diffusion or pressure differential

We assessed and optimized reporter gene transfection efficiency and persistence in collagen gel cultures (rat tail collagen type-I, 2–6 mg/ml) with the NP differential across 0.4 µm transwells. We examined the effect of NP level and collagen concentration (Figure S8), GET nanoparticle dose (Figure S9), and the positioning of the therapy in atmospheric- or NP- side with exposure for up to 24 h, collecting the media from both ambient (bottom) and NP (top) sides. Using single transfection of pGluc in any collagen concentration at ambient (passive diffusion) or pressure differential we achieved poor transfection levels (Figure S8). Applying NP on the side of the transfection or assaying the opposite side to the transfection exposure generated much lower transfection levels. This is likely to be due to the transfection being actively prevented from diffusion into the collagen by NP, and the Gluc reporter actively being drawn to the NP differential, respectively. This was also likely to be confounded by aggregation and collection of the nanoparticles on the porous membrane due to their low size and low diffusion even with NP*.* Charged particles rapidly aggregate in physiological ionic environments [57]. It is known that over time GET-pDNA in complex microenvironments tend to aggregate from smaller (~ 90 nm) to very large (> 1 µm) complexes [17]. Even high (10X) dosages did not dramatically improve transfection levels (Figure S9), and this was lower than if transfection was simply placed on top of collected from pre-transfected collagen gels (Figure S10), meaning this was not due to diffusion of the Gluc reporter. We hypothesized that pore size and density used in the 0.4 µm transwell set up could have negative influence on GET nanoparticles accessing the hydrogel. We used fluorescently labelled pDNA (TM-Rhodamine-label) packaged either into GET-pDNA complexes or as naked DNA (Figure S11) and compared the transfer of fluorescence across the transwells using NP. It was clear that neither naked nor packaged DNA was effectively transferred within the liquid that was transferred across the membrane by the differential. With the fluorescent signal accumulating on the ambient side of the membrane with NP application. This could be possibly due to particles clogging the transwell pores or aggregating when interacting with the membrane (Figure S11). There was very little that transferred or accumulated on the lower surface without a pressure differential, meaning that diffusion through the membrane was low. We used fluorescent versions of the GET peptide (TM-FLR) to assess if the peptide (~ 5 kDa) which is much smaller than pDNA (> 1mDa) of GET-pDNA complexes could transfer across the membrane (Figure S12). Labelled TM-FLR was as effective at transferring across the membrane (Figure S12A) as was 5.5 kDa Dextran (TRITC-labelled) (Figure S12B) with a pressure differential meaning that whether packed into nanoparticles or not, that pDNA could not be efficiently transferred across the 0.4 µm PET transwell membrane.

**Fig. 3:**
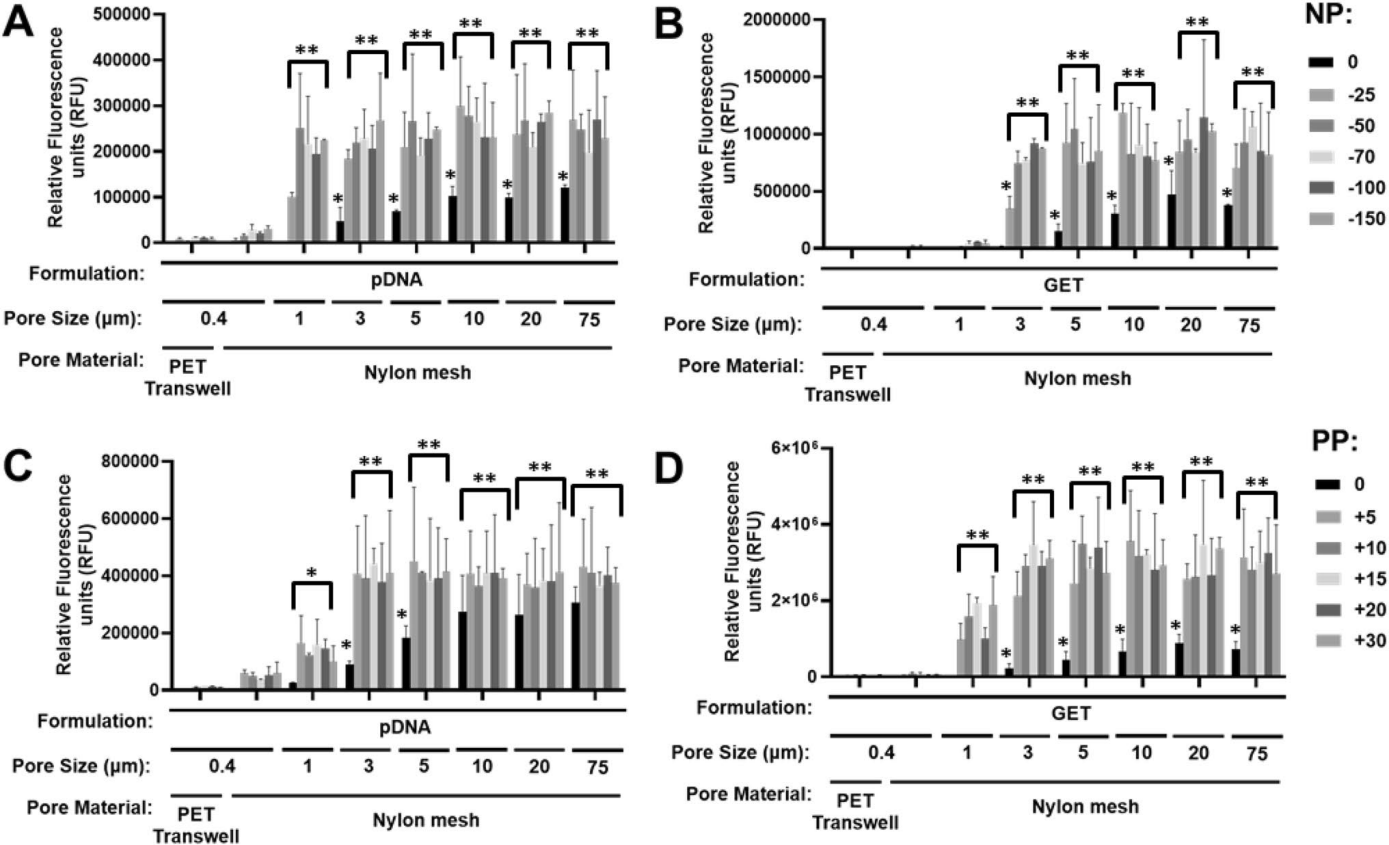
GET nanoparticles require multi-micron pores for effective diffusion with or without pressure differentials. **A**-**B** Fluorometry assay of **A**) Rhodamine-labelled pDNA (pDNA) or **B**) GET nanoparticles (generated using FLR:FLH and Rhodamine-labelled pDNA) transfected from bottom and measured from top media locations with NP differential (0 to −150 mmHg). **C** and **D** are repeats of A) and B) with PP differential (0 to + 30 mmHg) transfected from the top and measured from the bottom media location using 0.4 µm (PET) and 0.4–75 µm pore-size nylon mesh wells. (*N* = 6, bars are S.D. ** *p* < 0.01, * *p* < 0.05)

To overcome the pore-size issue with our system, we moved to generate bespoke transwells which could contain different pore sizes to assess if larger pores could improve transfer and ultimately transfection efficiency across the pressure differential (Fig. 2C). We obtained nylon meshes with pore sizes of 0.4–75 µm to test for transfer either by diffusion or using pressure differentials. Employing O-rings and transwells with the original membrane removed we created samples that were pressure-tight except through the pores and could be used to compare transfer. We confirmed that pore size was a significant block to transfer of pDNA through the membrane (Fig. 3). Even 0.4 µm nylon mesh improved transfer by diffusion or by using NP over the original 0.4 µm PET transwell membrane, although this was still restricted. We confirmed that > 3 µm was unrestrictive for both diffusion and using NP (Fig. 3A-B). PP was also examined using the same assays. We were able to demonstrate the exact opposite effect using PP differential (using low PP due to the set up; 15 mmHg). GET nanoparticles were not transferred effectively in 0.4 µm transwells under PP but were with bigger pore sizes (Fig. 3C-D) as for NP. Interestingly it is well known that collagen gels [58] and native soft-tissue [59] can have small matrix pore sizes of 0.5–10 µm (2 mg/ml collagen gel is ~ 2–4 µm) meaning alignment of the mesh and tissue porosity in our assays. We employed 3 µm pore membranes for further experiments as they were technically easier to use with collagen gels without leaking during gel setting.

### Cell capturing of GET nanoparticles by pressure differential

**Fig. 4:**
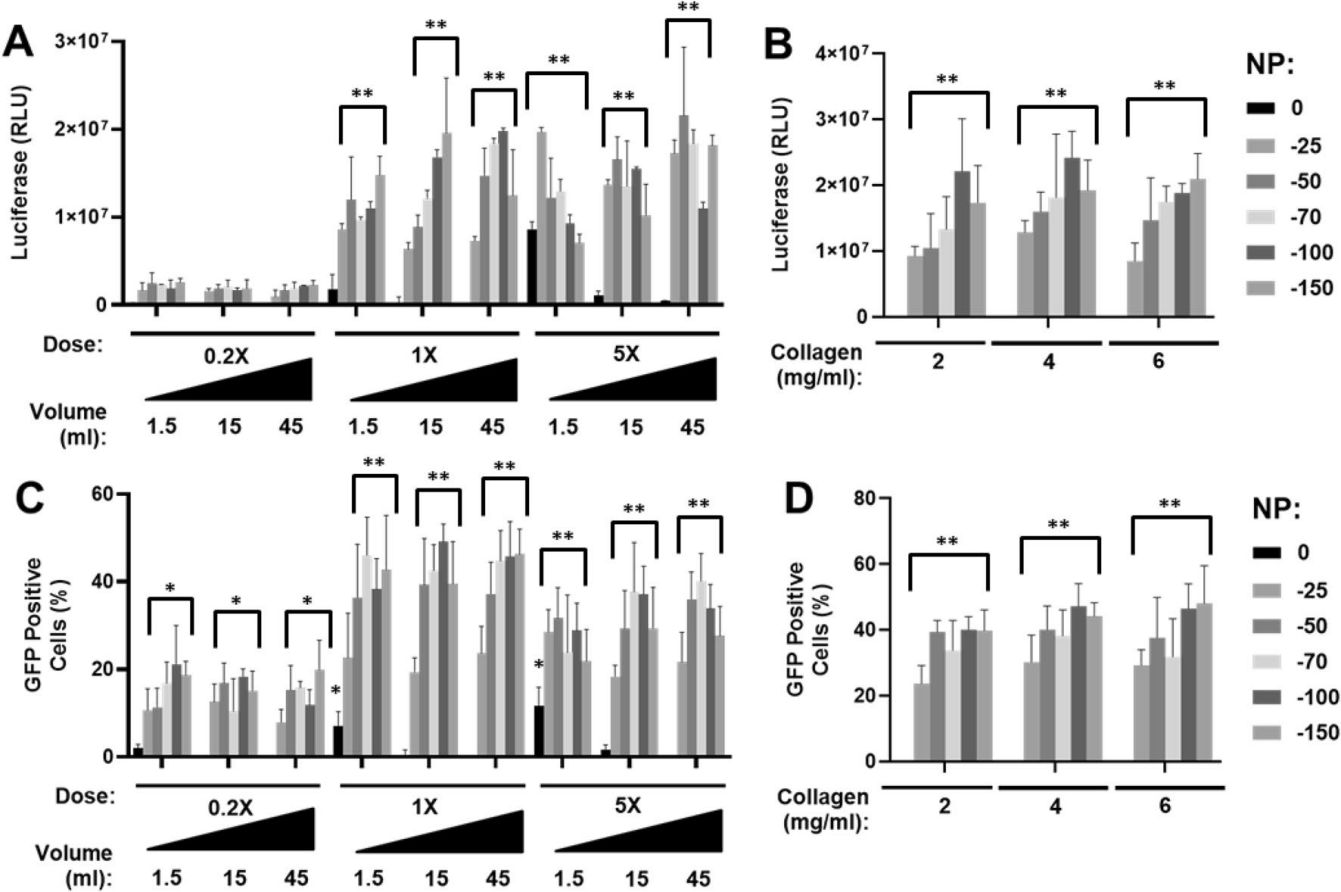
Dose, volume and hydrogel density transfection inhibition can be overcome with NP differential. **A** Luciferase assay of GET-pGluc nanoparticles (generated using FLR:FLH) transfected from bottom in different media volumes (1.5, 15, 45 ml) at different dosages (0.2X, 1,X 5X) in NIH3t3 (5 × 10^5^)-laden collagen (2 mg/ml) hydrogels. **B** Assessment of different collagen concentrations (2, 4, 6 mg/ml) with 1.5 ml volume and 1X dose. **C** GFP flow cytometry positivity (%) with delivery as in A, but delivering peGFP. **D** Assessment of different collagen concentrations as for B) with GFP transfection. After pressure application media replaced (0.5 ml top, 1.5 ml bottom) and luciferase measured from top media locations, GFP measured by flow cytometry in collagenase-recovered cells, and metabolic activity measured after 24 h. NP differentials (0 to −150 mmHg) using 3 µm pore-size nylon mesh wells (*N* = 6, bars are S.D. ** *p* < 0.01, * *p* < 0.05)

Using passive diffusion, placing the transfection in the top or the bottom well, the transfection efficiency was like that of simple transfection of collagen gels (when controlling for volume). We used NP to draw all liquid volume from the lower well through the collagen gel, transferred the samples to wells without GET-pDNA complexes and assessed if this ‘complete’ exposure to all formulation volume enhanced transfection (Fig. 4); indeed, this significantly enhanced transfection efficiency with even low NP levels. This effect appeared to be more dependent on the total amount of volume drawn through the sample rather than at what pressure was employed. In fact, lower pressures seemed to be optimal, and when confirming non-bypassing of the sample by the transferred liquid, data was more consistent in showing higher transfection levels with a slower transfer (Fig. 4A,C; Figure S13 for metabolic activity). The time to draw low volumes through the gels was dependent on the gel concentration and the pressure applied as expected. Gel concentration had lower effect on transfection efficiency when pressure differentials were used (Fig. 4B, D; Figure S14 for metabolic activity). As GET-pDNA appeared to be concentrated into the collagen gels with NP we conducted dilution experiments in which a fixed dose of therapy (0.2, 1 and 5X) was drawn through the sample delivered in different volumes. As expected, dilution of the therapy and relying on diffusion into the gel significantly lowered the transfection levels, however dilution had minimal effect if the therapy was drawn through the sample before culturing (Fig. 4A). It was vital to normalize the luciferase activity to the volume due to differential dilution of the reporter signal in larger delivery/collection volumes. GFP transfection aligned with Gluc luciferase data showing that dilution had minimal effect if the volume was drawn through the sample (Fig. 4C).

To determine the mechanism by which the volume, but not overall dose, appeared most important for transfection we conducted experiments using fluorescent variants and assessed fluorescence in delivery media and extracted collagen. We compared these to levels post-collagen transfer in received media, to confirm if media was being depleted of GET-pDNA by fluid-flow through collagen (Figure S15). We were able to confirm ‘filtering out’ of pDNA associated with the GET peptide (Figure S15A, B), and the FLR peptide alone (Figure S15C, D) specifically in cell-loaded gels, but not significant filtering of naked pDNA. We repeated experiments with cell-free and cell-loaded fixed gels, and this filtering was dramatically lower in cell-free gels but remained high in paraformaldehyde (PFA) fixed cell-laden gels suggesting that cells (viable or not) could interact with and sequestering the GET peptide or its associated nanocomplexes as they were drawn through the collagen gel.

We repeated these experiments with GET inhibitor Heparin [10] which competes for GET-binding with cells (Figure S16). As previously shown for conventional culture transfections, Heparin could prevent this filtering by inhibiting the binding of GET-pDNA to cells (Figure S16A, B). To confirm cell-mediated filtering of the GET-pDNA we assessed the effect of cell concentration, we demonstrated that cell density placed a significant role in this phenomenon, with higher numbers of cells being able to sequester more peptide than lower levels in an almost linear fashion (Figure S16C, D). The data confirms that either cell number or GET-pDNA concentration can be limiting in this system, meaning that in even dense cell-laden gels very high concentrations of GET-pDNA could be captured in collagen gels by cells.

**Fig. 5:**
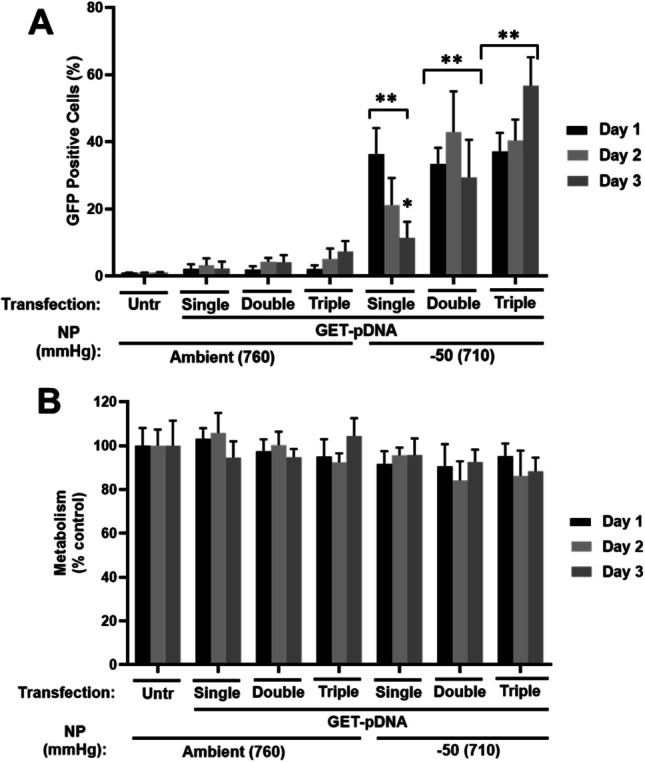
Serial short-exposure dosing as a variable in transfection of GFP pDNA with NP differential and GET nanoparticles. **A** Flow cytometry quantification of cells transfected with peGFP GET nanoparticles (generated using FLR:FLH), delivered from bottom for 30 min once (Single, Day0), twice (Double, Day0 & 1) or three-times (Triple, Day0, 1 & 2) to NIH3t3 collagen gels measured at day 1–3 (1 µg/ml.). **B** Metabolic activity (PrestoBlue) of NIH3t3 cell collagen hydrogels measured at Day 1–3. Data was normalized to untransfected (Untr) cells in either monolayer or collagen hydrogel formats as 100%. (*N* = 6, bars are S.D. ** *p* < 0.01, * *p* < 0.05)

Serial deliveries to collagen could be achieved by using moderate pressure differential and we could load samples with GET-pDNA by drawing formulations through them with NP daily after reading previous day transfection and viability levels (Fig. 5). We used a regimen that required only 30 min of connection to the system (45 ml containing a 1X dose at 50 mmHg) meaning that a minimal (30 min missing collection) was lost during transfection. This significantly enhanced the longevity of expression and number of transfected cells on day 3 (~ 10% to ~ 58% with single [Day 0] or daily [triple, Day 0, 1 and 2] deliveries measured at Day 2). We therefore conclude that using a pressure differential is amenable to high volume, low dose samples. These can be applied serially to cells in tissue-like matrices to achieve the highest gene transfer efficiency.

### Focusing gene therapy at the site of pressure-differential

**Fig. 6:**
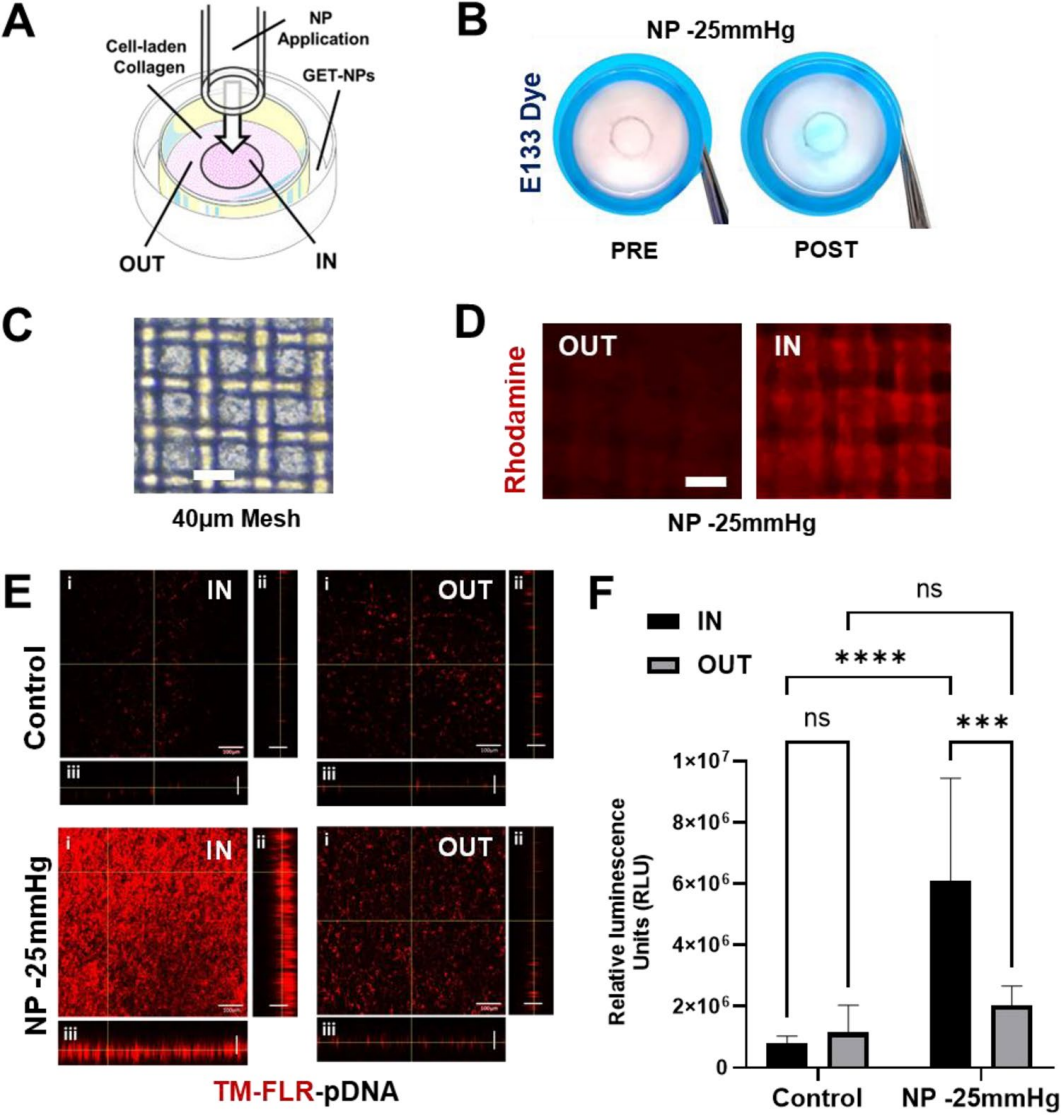
Negative pressure-mediated localized delivery. **A** Schematic of the 3D *in-vitro* model assembly of collagen-engineered tissue in a cell strainer to allow localized NP to be administered A 40 µm-pore size cell strainer with 500µL NIH3t3 cell-laden collagen hydrogels (1 × 10^7^ cells/mL), plated on the outer side of the strainer. Hydrogels were left to set for 30 min at 37 °C, 5% CO_2_ then the cell strainers were flipped upright to fit a 6-well plate filled with 1 mL warm growth media. NP (−25 mmHg) was applied by the RENASYS NPWT system fitted with a 6 mm plastic tubing placed inside the strainer on a central 6 mm circle (IN area). **B** Acellular collagen hydrogels pre- and post NP application (−25 mmHg for 1 min) on the central IN circle with E133 dye (Acid Blue 9/Erioglaucine disodium salt, 10 μM) in the bottom compartment showing localised fluid flow. **C** Microscopy of cellularized collagen hydrogels (bar 40 µm). **D** Demonstration with fluorescence microscopy of Rhodamine distribution in IN and OUT areas following NP application as in B (bar 40 µm). **E** Localization of GET-pDNA (with labelled FLR, TM-FLR) after NP application (as in B) in cellularized collagen hydrogels. This shows high localization of GET-NPs in IN region, and poor delivery in controls (bars: i, 100 µm; ii-iii, 500 µm). **F** Localized NP-mediated transfection. GET-pGluc nanoparticles (10 µg pDNA) were added in the bottom compartment then 5 min NP (−25 mmHg) applied. The IN area was removed by biopsy punch and both IN and OUT samples transferred to fresh media at day 1 post-transfection for extra 24 h to record luminescence from defined areas (*N* = 3 in triplicates, bars are S.D. **** *p* < 0.0001, *** *p* < 0.0002)

As pressure-differential has such significant effect on cell-interaction of GET-pDNA complexes and therefore transfection we aimed to assess if this focusing of fluid-flow affects adjacent tissue areas not under pressure differential. We had already tested transfecting from the ‘wrong side’ of the pressure differential (Figure S17). Next, we more closely assessed if NP application to draw away transfection could inhibit that achieved by ambient pressure diffusion. This was interesting as the fluid flow was placed against diffusion of GET-pDNA into the sample and this yielded a reduction in the lower levels of transfection seen by conventional simple incubation (same side ~ 4.3-fold reduction under −25 mmHg NP). We next conducted experiments where only a small area of the collagen hydrogel was exposed to NP by using lower diameter tubing, directly applied to the surface of the gel in larger netwells (40 µm pores) (Fig. 6A). These experiments were technically challenging aiming not to break and therefore bypass the gel; however, this was achieved for short 5 min NP exposures. Initially we aimed to confirm the location of the fluid flow generated using a rapid and visual method. We tested E133 blue (Acid Blue 9/Erioglaucine)-dye transfer which we have previously used as a cytocompatible marker of GET-transfection transfer from hydrogels (Pluronic/Methyl cellulose; [16]). E133 was added to the delivery media (10µM) and assessed the progressive transfer visually (Fig. 6B). At low NP (−25 mmHg) we demonstrated rapid transfer (5 min) to the applied area (termed as IN) and little in adjacent areas (termed OUT). Longer exposures (> 15 min) lead to diffusion into the gel and drawing into the collagen in non-applied areas. We confirmed this localization could be microscopically imaged using Rhodamine dye (Fig. 6C, D).

To quantitatively confirm targeting by NP of GET we tested the direct transfer of GET-pDNA from delivery media to cells using -labelled TM-FLR (Fig. 6E). It was clear by microscopy and fluorometry (Figure S18) that GET-pDNA transferred with highly specificity to the IN (NP-applied collagen) region over the OUT region. Next, to demonstrate fluid-flow enhanced transfection and direct transfer of GET-pDNA from delivery media to cells residing in collagen, we examined whether the applied area ‘IN’ had enhanced transfection (Fig. 6F). We delivered GET-pDNA in the delivery media for different durations (at −25 mmHg), excised NP applied area (IN) away from non-applied areas (OUT), washed them and incubated them for 24 h to measure the transfection efficiency (similar to previous targeted transfection analyses [16, 50, 60]). It was clear that positioning of the NP promoted the direct transfection of the cells in the IN region. When comparing whole sample verses focused-application of NP there was specificity in the IN-area (~ sixfold) for transfection efficiency over the OUT area (black bars, Fig. 6F). Interestingly, the miss-targeting of gene transfer in the OUT-area versus delivery to both IN/OUT areas was actually increased by the overall NP, which is likely due to fluid-flow from media, via OUT to IN regions, drawing in GET-pDNA complexes from outside the focused area (grey bars, Fig. 6F).

### Enhanced gene therapy in ex vivo skin with negative pressure

**Fig. 7:**
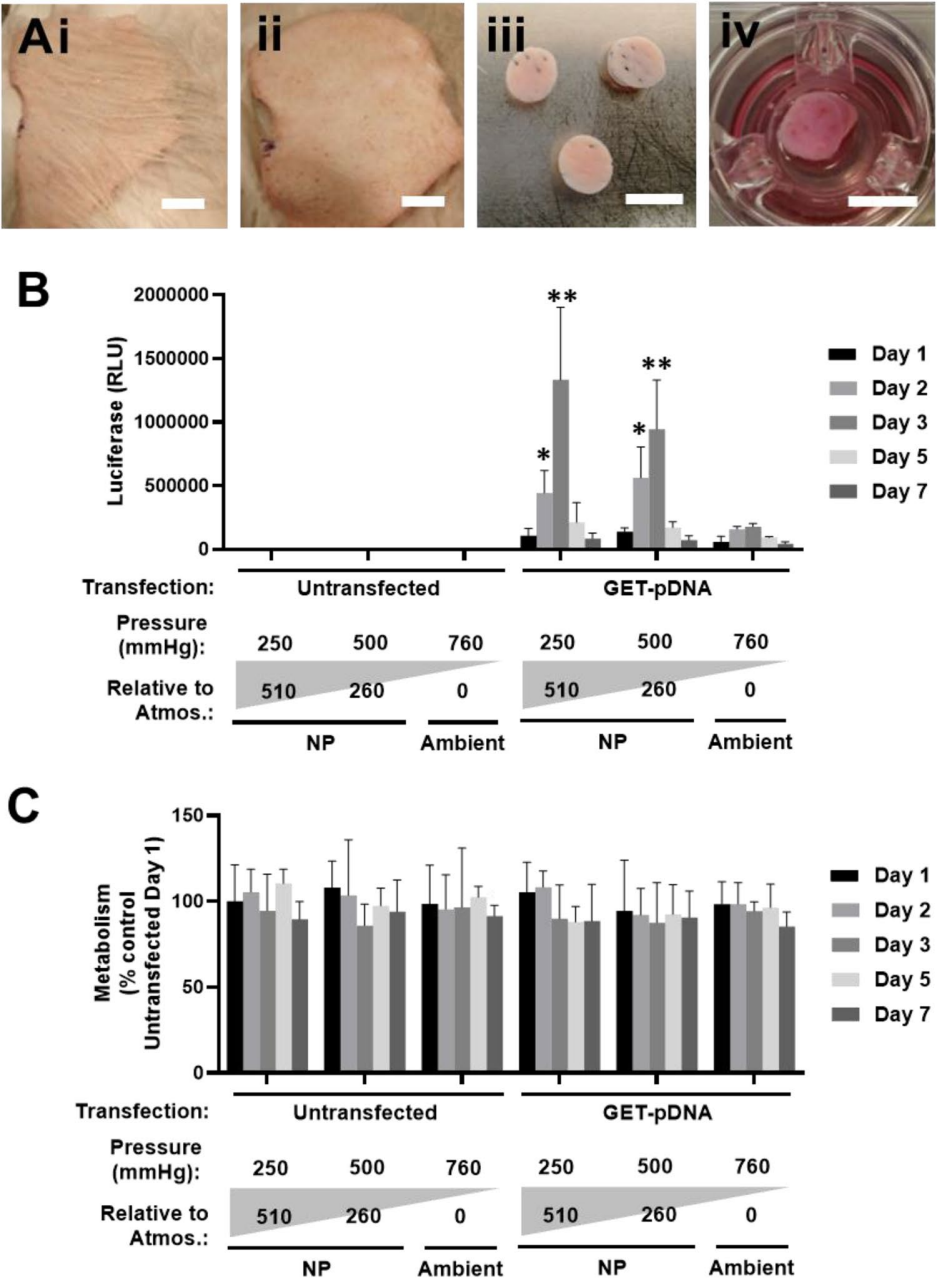
Negative pressure-mediated transfection of pig skin explants ex vivo. **A** Processing of pig skin for explant culture. i. Fresh skin sheet, ii. Shaved and cleaned skin sheet. iii. 8 mm biopsy punched explants. iv. Transwells with explants (bars: i-ii, 15 mm; iii-iv, 10 mm). **B** Luciferase assay of GET-pGluc nanoparticles (generated using FLR:FLH) transfected from bottom (4 µg pDNA dose) for 24 h, measured at day 1–7 post-transfection. Ambient and NP differentials (0, −250 and −500 mmHg) with 3 µm pore-size nylon mesh wells were used (*N* = 6, bars are S.D. ** *p* < 0.01, * *p* < 0.05). **C** Metabolic activity (PrestoBlue) experiment in B. Data was normalized to untransfected explants as 100%. (*N* = 6, bars are S.D. ** *p* < 0.01, * *p* < 0.05)

As a complete validation of pressure-mediated transfection using GET, we transfected explants of viable pig skin (Fig. 7A). GET was shown to mediate gene transfer at ambient pressures with dose important for level and duration of transgene expression (Figure S19). Application of NPWT using the transwell system (tissue plugged with agarose) demonstrated enhanced transfection level and duration using the clinical system (Fig. 7B) without an effect on tissue viability by metabolic assay (Fig. 7C). Therefore, NP may enhance transfection and does not inhibit gene therapy to native skin when tested ex vivo.

## Discussion

### Negative pressure to direct GET gene therapy in tissues

Inspired by previous work on pressure-mediated transfection of naked DNA and the increasing application of NPWT to treat wounds, we were prompted to integrate our non-viral gene delivery system, GET, with pressure to enhance the distribution, efficacy of expression and biological effect on target-tissue. Nanoparticle bioadhesion is important for tissue diffusion and for cell interaction and uptake. We have previously generated more highly diffusible GET formulations using PEGylation to control particle charge but retain function [17]. Work here, however, suggests that if diffusion is to be expected on millimetre scales then simple molecular engineering of the delivery system will not circumvent the bottle-neck. Active transport through thick tissues either by hijacking perfusion with systemic delivery or by drawing the formulation through tissue by another means is vital for full distribution into the target tissue. Application of reasonably low amounts of NP to tissue can mediate fluid-flow, and this is much more influential on full tissue delivery than the bioadhesiveness of the system.

To apply NP across a tissue for fluid-flow we devised an innovative cell culture system to use clinical NPWT machinery (and Insufflators for PP) allowing application of pressure differentials across cells using an engineered tissue collagen hydrogel system. This retained cell viability and allowed both PP and NP to be applied to cells in culture. Cells retained moisture, temperature, nutrient, and gaseous exchange within the system vital for successful in vitro cell culture.

Applying fluid-flow approaches to enhance delivery to tissue is not only applicable to skin. This approach would be valuable for a number of disorders to allow therapeutic gene expression in a controlled manner, at least to external tissue layers such as the skin, eye or internally such as intestine. Our approach therefore allows targeting of nucleic acid therapeutics, and development of systems directing healing, potentially using transient expression of cytokines or growth factors with genes to promote regeneration.

### Pressure-directed controlled delivery of GET gene therapy

We carefully dissected the dynamics of GET-pDNA gene delivery in the context of NP application in our tissue systems. We could determine the levels of transfer of GET-pDNA from the delivery media into the tissue, and subsequent internalization of complexes into cells. Short exposures (5 min) focused on a defined area of hydrogel allowed us to use NPWT system to regionally transfer GET-pDNA to cells in 3D without affecting metabolism or viability. During this time there was minimal uptake, however cell membrane binding in the period subsequently led to uptake and transgene expression. This meant that even with this short exposure we could focus gene transfer to cells directly in collagen gel regions that were undergoing NP-induced fluid-flow. Equally importantly the reciprocal was also evident, demonstrating the approach can limit non-pressure applied cell gene transfer (decrease in reporter levels in non-targeted cells) in the same tissue (Fig. 6).

Interestingly, although targeted areas of hydrogel (IN samples) had the most significantly enhanced transfection with NP, OUT areas also had significantly improved transfection, although much lower than the IN region (Fig. 6F). It is likely that fluid-flow is not completely faithful in the vertical plane, meaning that GET-pDNA could be drawn into the OUT region, through the hydrogel and into the adjacent region. Quantitation of TM-FLR localization in gels demonstrated that only samples with applied NP had enhanced distribution in all sample depths (0–500 µm) but also that the OUT region did have increased concentration of FLR with NP, this however being minimal compared to the IN region (Figure S18). Taken together it is clear that applying a pressure differential can allow the control of delivery of the GET gene therapy system into engineered collagen tissues. Transfection using a system that allows fluid to be drawn into the sample allows loading of the sample with poorly diffusing nanocomplexes and significant gene transfection which cannot be achieved by simple incubation.

### Cell membrane binding with subsequent uptake is important for pressure-mediated transfection

Previous studies have demonstrated that GET peptides interact very effectively with cell membranes and are rapidly internalized. The turnover of heparan sulphate on the cell membrane is required to recycle the capacity of new interactions with more GET peptide. This is very important in the context of normal dosages of GET-pDNA complexes. It was interesting to observe that at higher dosages of nanocomplexes per cell, either providing: 1) a higher cell number, or 2) slower delivery (i.e., lower pressure differential) were more impactful in allowing the capture of all GET-pDNA that was perfused (Fig. 4, 6). At the highest levels of NP (−150 mmHg) there was a slight decrease in cell viability (Figure S11, S12), considering the amount and rapidity of fluid drawn through the sample this is not surprising. Importantly, although such pressures are used clinically on wounded tissue, these are biophysically much harder than tissue engineered from type-I collagen. Also, authentic tissue is heavily vascularized and thicker (physically and in terms of ECM density) meaning that the direct pressure that is placed on native cells its likely to be much lower in tissue than our collagen engineered tissue system. Viable pig skin, cultured ex vivo, was demonstrated to be readily transfectable but at low levels (Figure S19). Using pressure differential transfection was enhanced in terms of level and duration of transgene expression using negative pressure without affecting viability, similarly to collagen hydrogels (Fig. 7). This shows that clinically deployed NPWT approaches can enhance gene therapy in the context of authentic skin.

## Conclusion

Directed gene delivery to sites in tissue, such as skin as an intradermal constituent of wound healing strategies, could be transformative in traumas such as physical damage or burns, and in conditions such as dermatitis or skin cancers. Therapies to the eye or gut could also be translated using our approach. Employing non-viral gene delivery in a tractable format to aid regenerative medicine approaches, including gene therapies could have immense impact for a number of disorders. The ability to simply deliver genes to specific regions of tissue by applying NP to the tissue, using systems already in clinical standard-of-care, will facilitate new drug delivery strategies and allow approaches to site-specifically deliver the activity of novel nanotechnologies and gene therapeutics.

## Supplementary Information

Below is the link to the electronic supplementary material.

Supplementary file1 (PPTX 969 KB)

## Data Availability

Data and materials are available on request.
