# Supplementary material for: Enhanced localized pressure-mediated non-viral gene delivery
Source: Drug Deliv Transl Res. 2025 Mar 12;15(10):3679–94. doi: 10.1007/s13346-025-01827-7 (PMC12397159; doi:10.1007/s13346-025-01827-7)
Supplement: Supplementary file 1 — Supplementary file1 (PPTX 969 KB) [file 13346_2025_1827_MOESM1_ESM.pptx]

## Slide 1
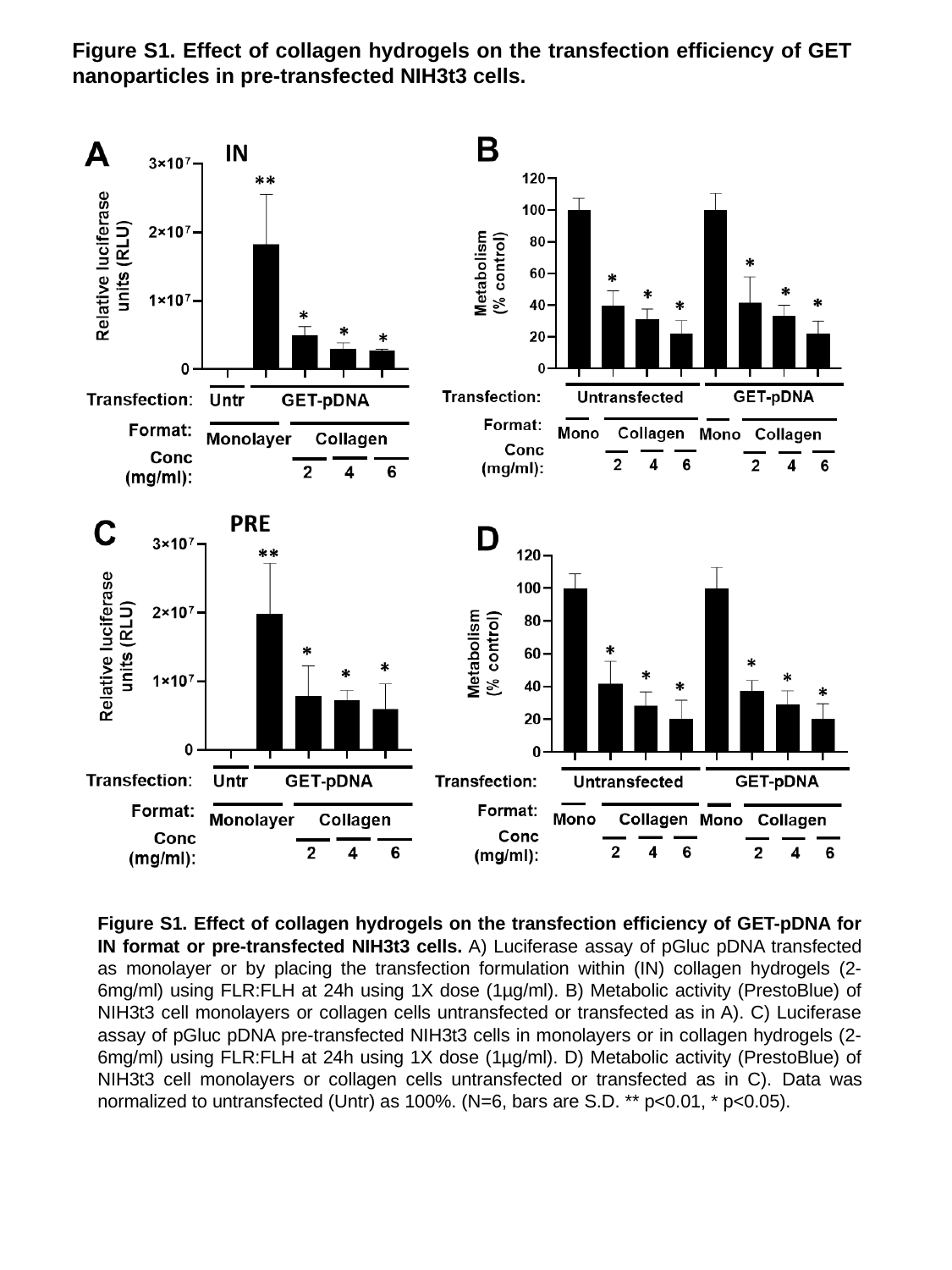

Figure S1. Effect of collagen hydrogels on the transfection efficiency of GET nanoparticles in pre-transfected NIH3t3 cells.
Figure S1. Effect of collagen hydrogels on the transfection efficiency of GET-pDNA for IN format or pre-transfected NIH3t3 cells. A) Luciferase assay of pGluc pDNA transfected as monolayer or by placing the transfection formulation within (IN) collagen hydrogels (2-6mg/ml) using FLR:FLH at 24h using 1X dose (1µg/ml). B) Metabolic activity (PrestoBlue) of NIH3t3 cell monolayers or collagen cells untransfected or transfected as in A). C) Luciferase assay of pGluc pDNA pre-transfected NIH3t3 cells in monolayers or in collagen hydrogels (2-6mg/ml) using FLR:FLH at 24h using 1X dose (1µg/ml). D) Metabolic activity (PrestoBlue) of NIH3t3 cell monolayers or collagen cells untransfected or transfected as in C). Data was normalized to untransfected (Untr) as 100%. (N=6, bars are S.D. ** p<0.01, * p<0.05).

## Slide 2
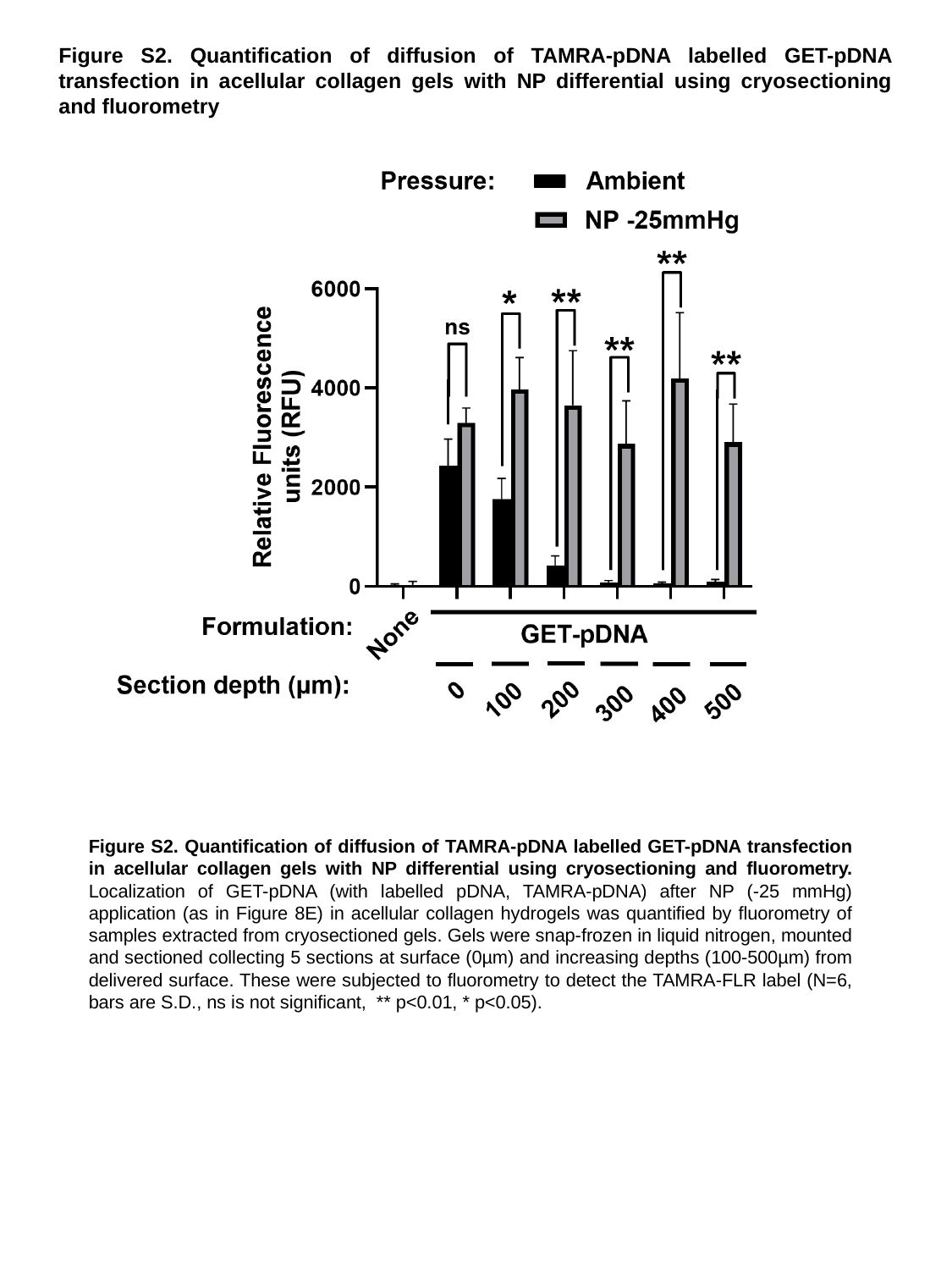

Figure S2. Quantification of diffusion of TAMRA-pDNA labelled GET-pDNA transfection in acellular collagen gels with NP differential using cryosectioning and fluorometry
Figure S2. Quantification of diffusion of TAMRA-pDNA labelled GET-pDNA transfection in acellular collagen gels with NP differential using cryosectioning and fluorometry. Localization of GET-pDNA (with labelled pDNA, TAMRA-pDNA) after NP (-25 mmHg) application (as in Figure 8E) in acellular collagen hydrogels was quantified by fluorometry of samples extracted from cryosectioned gels. Gels were snap-frozen in liquid nitrogen, mounted and sectioned collecting 5 sections at surface (0µm) and increasing depths (100-500µm) from delivered surface. These were subjected to fluorometry to detect the TAMRA-FLR label (N=6, bars are S.D., ns is not significant, ** p<0.01, * p<0.05).

## Slide 3
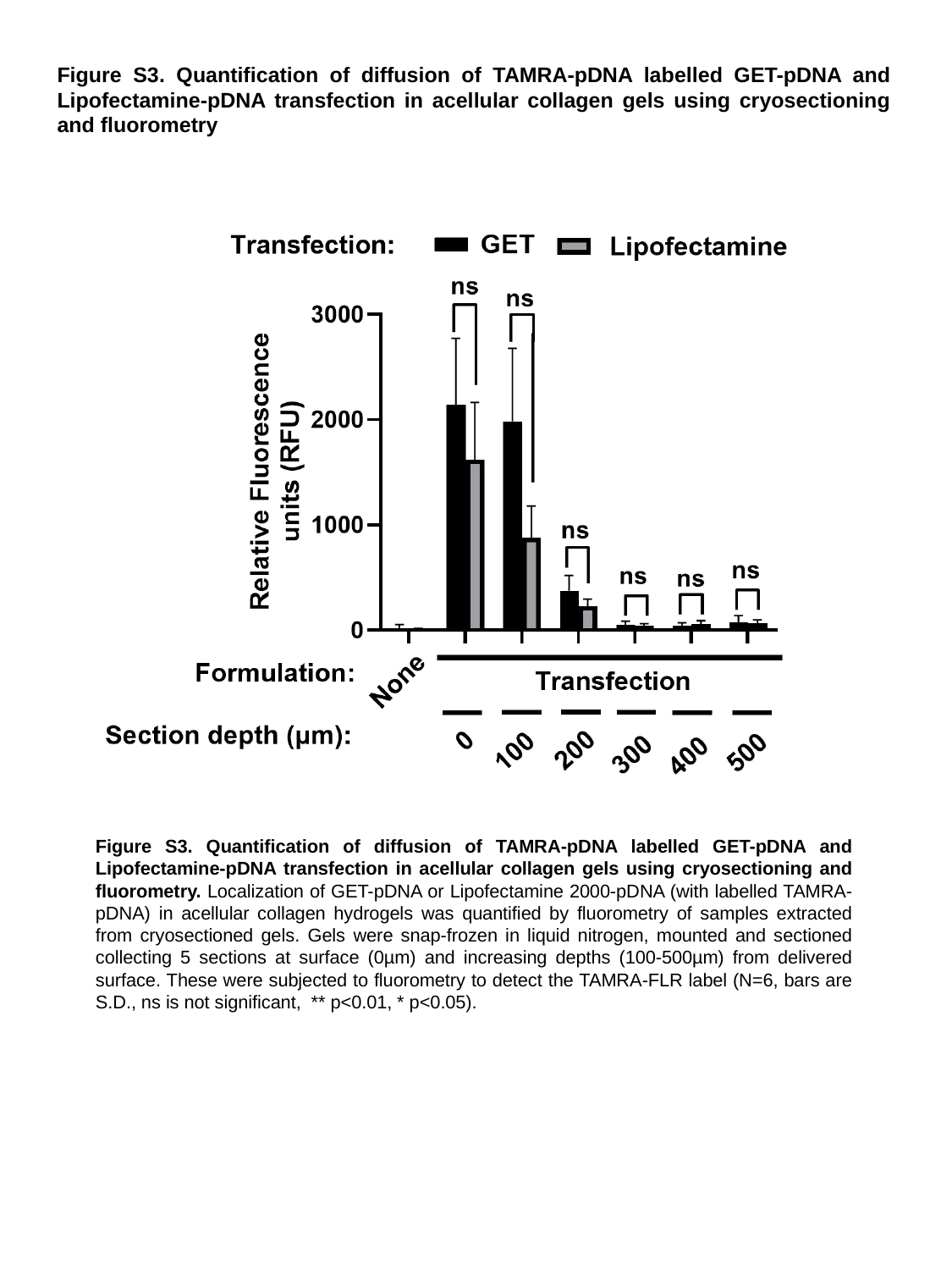

Figure S3. Quantification of diffusion of TAMRA-pDNA labelled GET-pDNA and Lipofectamine-pDNA transfection in acellular collagen gels using cryosectioning and fluorometry
Figure S3. Quantification of diffusion of TAMRA-pDNA labelled GET-pDNA and Lipofectamine-pDNA transfection in acellular collagen gels using cryosectioning and fluorometry. Localization of GET-pDNA or Lipofectamine 2000-pDNA (with labelled TAMRA-pDNA) in acellular collagen hydrogels was quantified by fluorometry of samples extracted from cryosectioned gels. Gels were snap-frozen in liquid nitrogen, mounted and sectioned collecting 5 sections at surface (0µm) and increasing depths (100-500µm) from delivered surface. These were subjected to fluorometry to detect the TAMRA-FLR label (N=6, bars are S.D., ns is not significant, ** p<0.01, * p<0.05).

## Slide 4
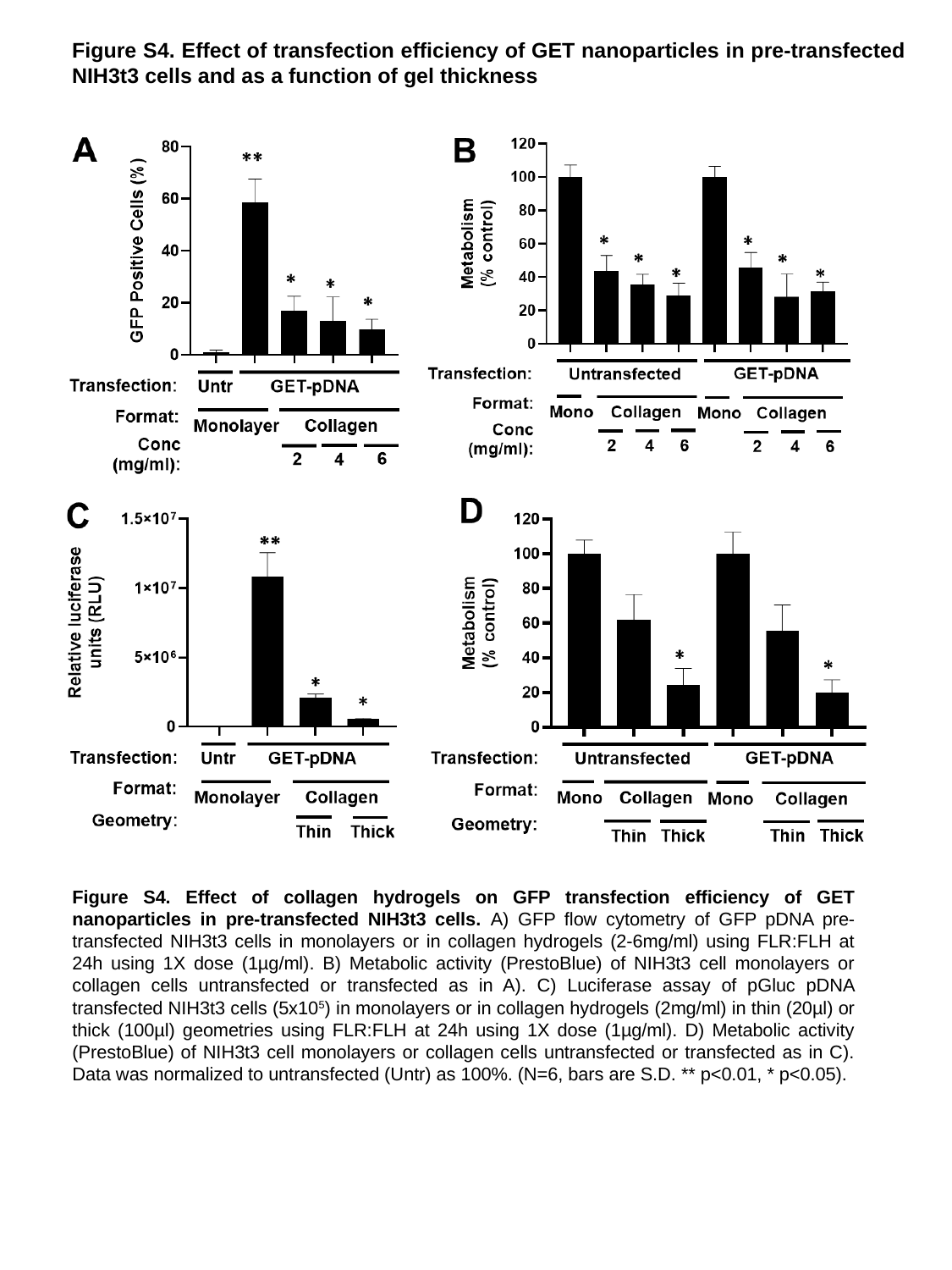

Figure S4. Effect of transfection efficiency of GET nanoparticles in pre-transfected NIH3t3 cells and as a function of gel thickness
Figure S4. Effect of collagen hydrogels on GFP transfection efficiency of GET nanoparticles in pre-transfected NIH3t3 cells. A) GFP flow cytometry of GFP pDNA pre-transfected NIH3t3 cells in monolayers or in collagen hydrogels (2-6mg/ml) using FLR:FLH at 24h using 1X dose (1µg/ml). B) Metabolic activity (PrestoBlue) of NIH3t3 cell monolayers or collagen cells untransfected or transfected as in A). C) Luciferase assay of pGluc pDNA transfected NIH3t3 cells (5x10^5^) in monolayers or in collagen hydrogels (2mg/ml) in thin (20µl) or thick (100µl) geometries using FLR:FLH at 24h using 1X dose (1µg/ml). D) Metabolic activity (PrestoBlue) of NIH3t3 cell monolayers or collagen cells untransfected or transfected as in C). Data was normalized to untransfected (Untr) as 100%. (N=6, bars are S.D. ** p<0.01, * p<0.05).

## Slide 5
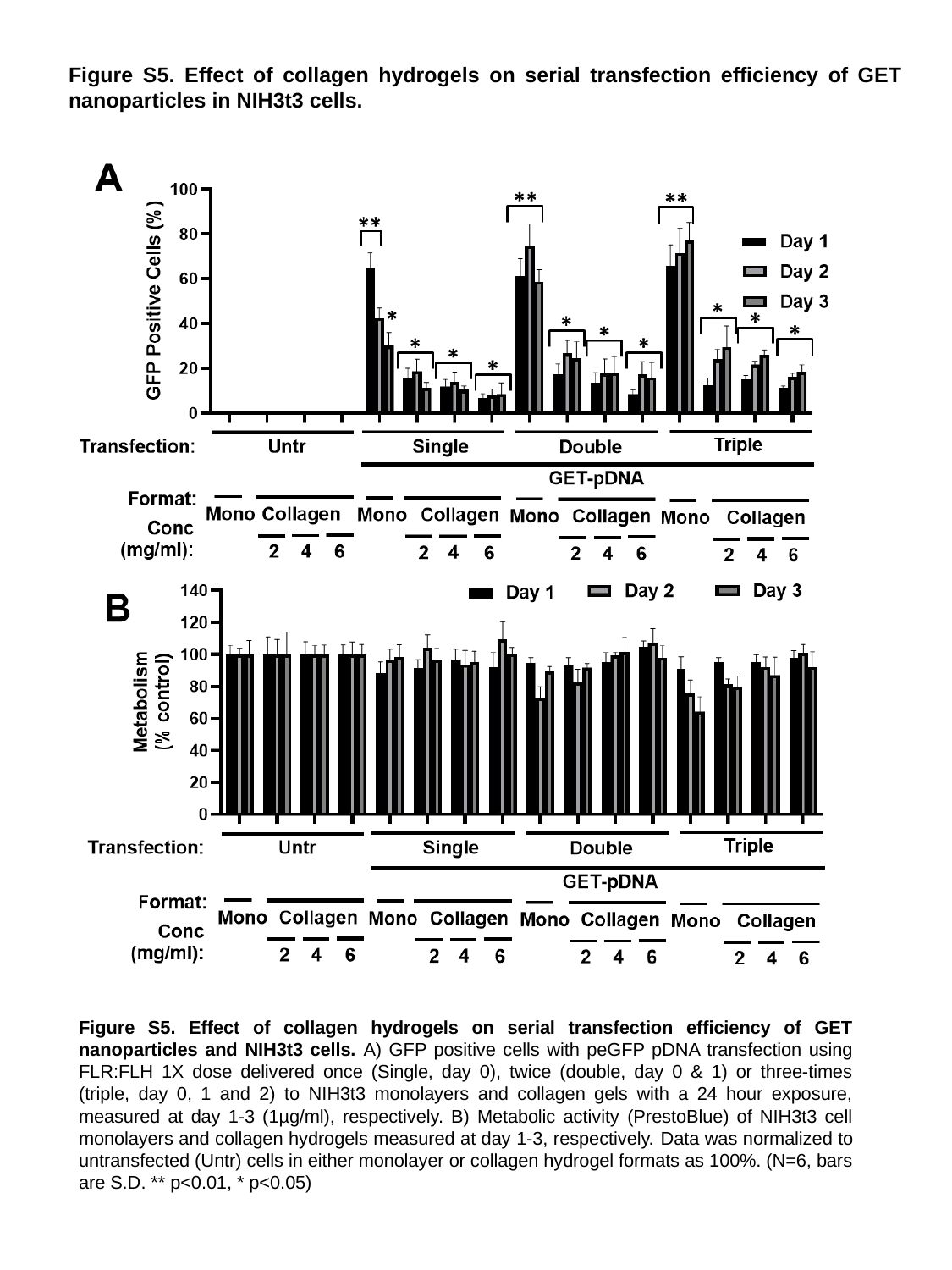

Figure S5. Effect of collagen hydrogels on serial transfection efficiency of GET nanoparticles in NIH3t3 cells.
Figure S5. Effect of collagen hydrogels on serial transfection efficiency of GET nanoparticles and NIH3t3 cells. A) GFP positive cells with peGFP pDNA transfection using FLR:FLH 1X dose delivered once (Single, day 0), twice (double, day 0 & 1) or three-times (triple, day 0, 1 and 2) to NIH3t3 monolayers and collagen gels with a 24 hour exposure, measured at day 1-3 (1µg/ml), respectively. B) Metabolic activity (PrestoBlue) of NIH3t3 cell monolayers and collagen hydrogels measured at day 1-3, respectively. Data was normalized to untransfected (Untr) cells in either monolayer or collagen hydrogel formats as 100%. (N=6, bars are S.D. ** p<0.01, * p<0.05)

## Slide 6
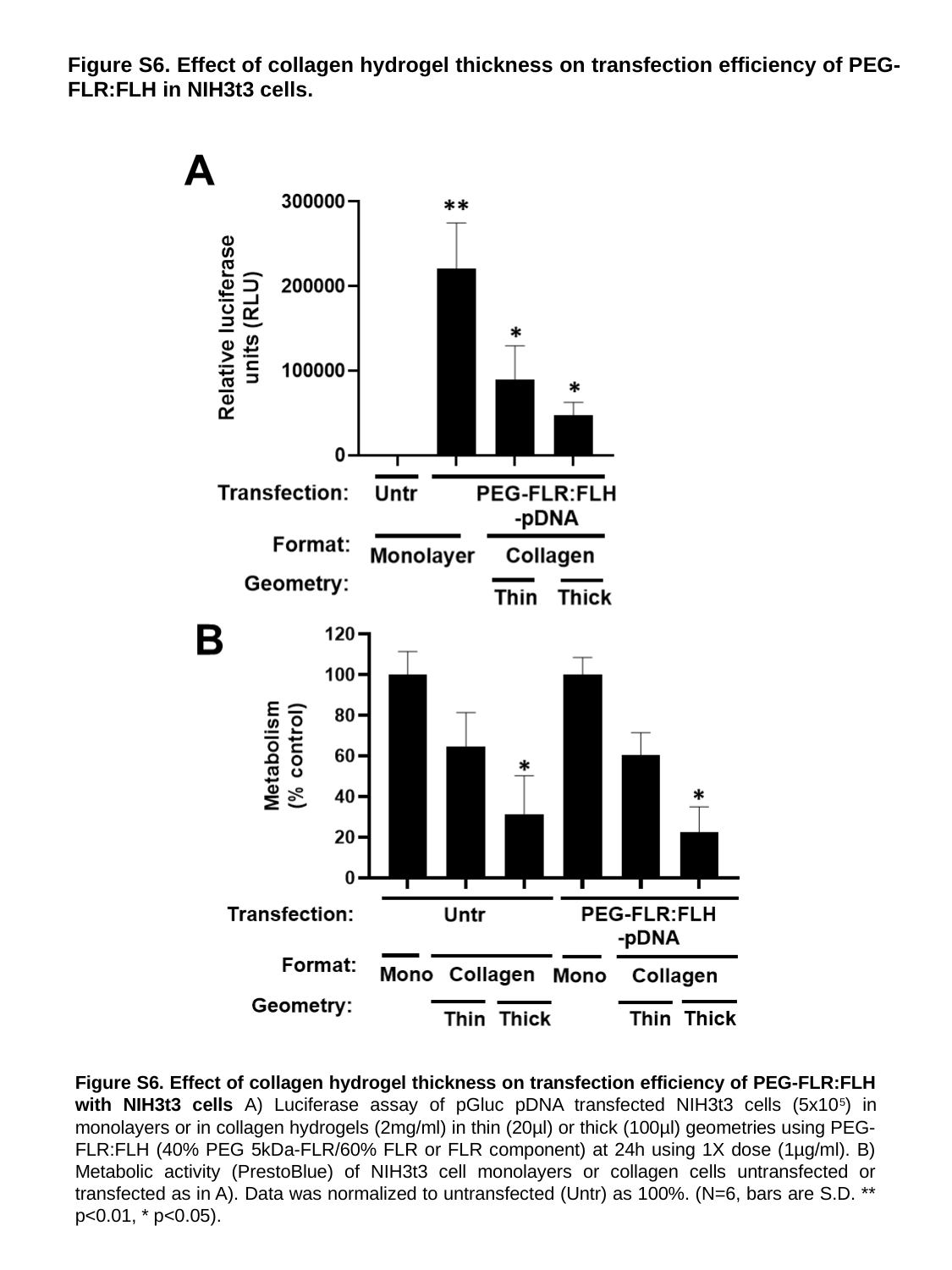

Figure S6. Effect of collagen hydrogel thickness on transfection efficiency of PEG-FLR:FLH in NIH3t3 cells.
Figure S6. Effect of collagen hydrogel thickness on transfection efficiency of PEG-FLR:FLH with NIH3t3 cells A) Luciferase assay of pGluc pDNA transfected NIH3t3 cells (5x10^5^) in monolayers or in collagen hydrogels (2mg/ml) in thin (20µl) or thick (100µl) geometries using PEG-FLR:FLH (40% PEG 5kDa-FLR/60% FLR or FLR component) at 24h using 1X dose (1µg/ml). B) Metabolic activity (PrestoBlue) of NIH3t3 cell monolayers or collagen cells untransfected or transfected as in A). Data was normalized to untransfected (Untr) as 100%. (N=6, bars are S.D. ** p<0.01, * p<0.05).

## Slide 7
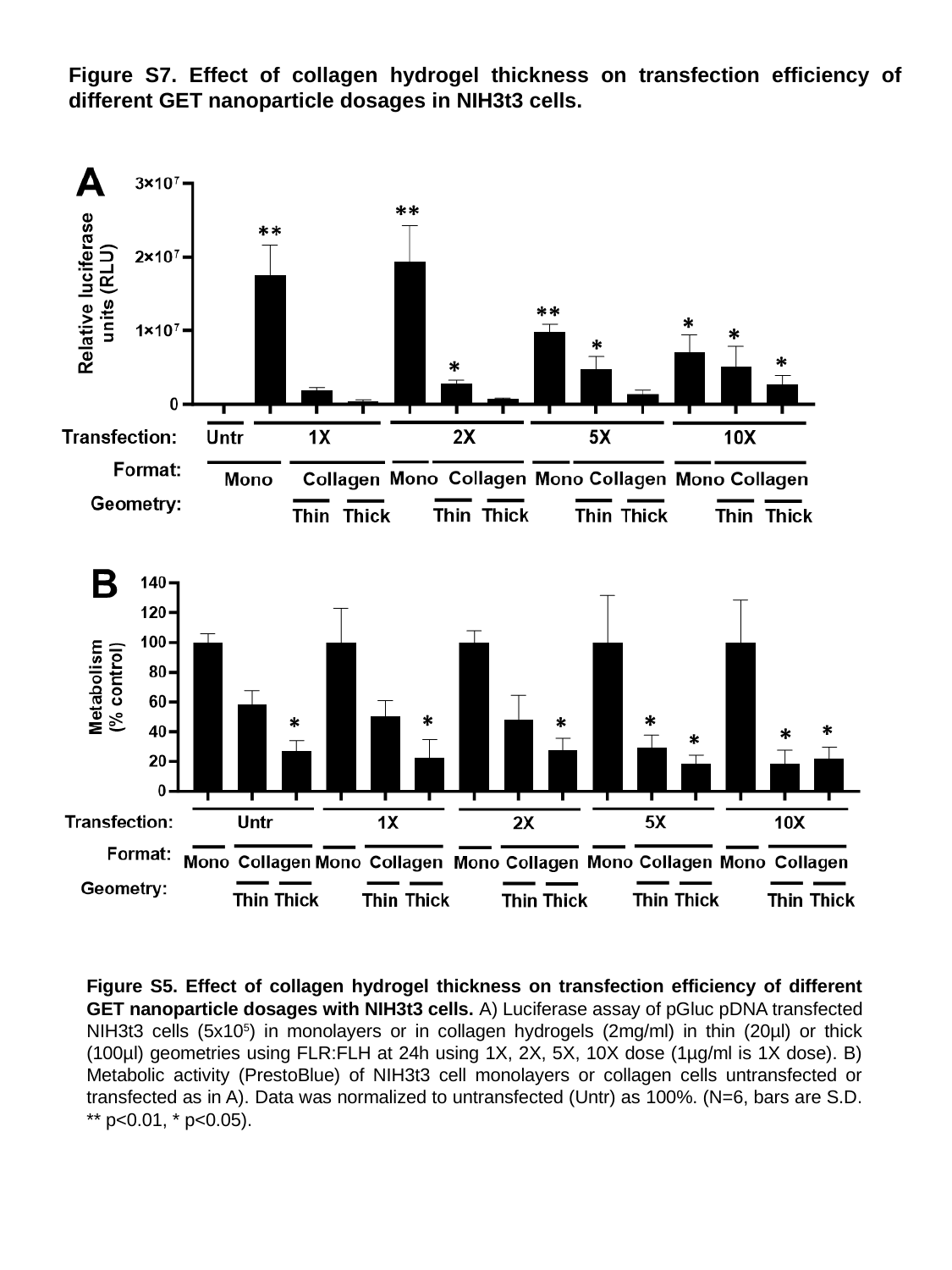

Figure S7. Effect of collagen hydrogel thickness on transfection efficiency of different GET nanoparticle dosages in NIH3t3 cells.
Figure S5. Effect of collagen hydrogel thickness on transfection efficiency of different GET nanoparticle dosages with NIH3t3 cells. A) Luciferase assay of pGluc pDNA transfected NIH3t3 cells (5x10^5^) in monolayers or in collagen hydrogels (2mg/ml) in thin (20µl) or thick (100µl) geometries using FLR:FLH at 24h using 1X, 2X, 5X, 10X dose (1µg/ml is 1X dose). B) Metabolic activity (PrestoBlue) of NIH3t3 cell monolayers or collagen cells untransfected or transfected as in A). Data was normalized to untransfected (Untr) as 100%. (N=6, bars are S.D. ** p<0.01, * p<0.05).

## Slide 8
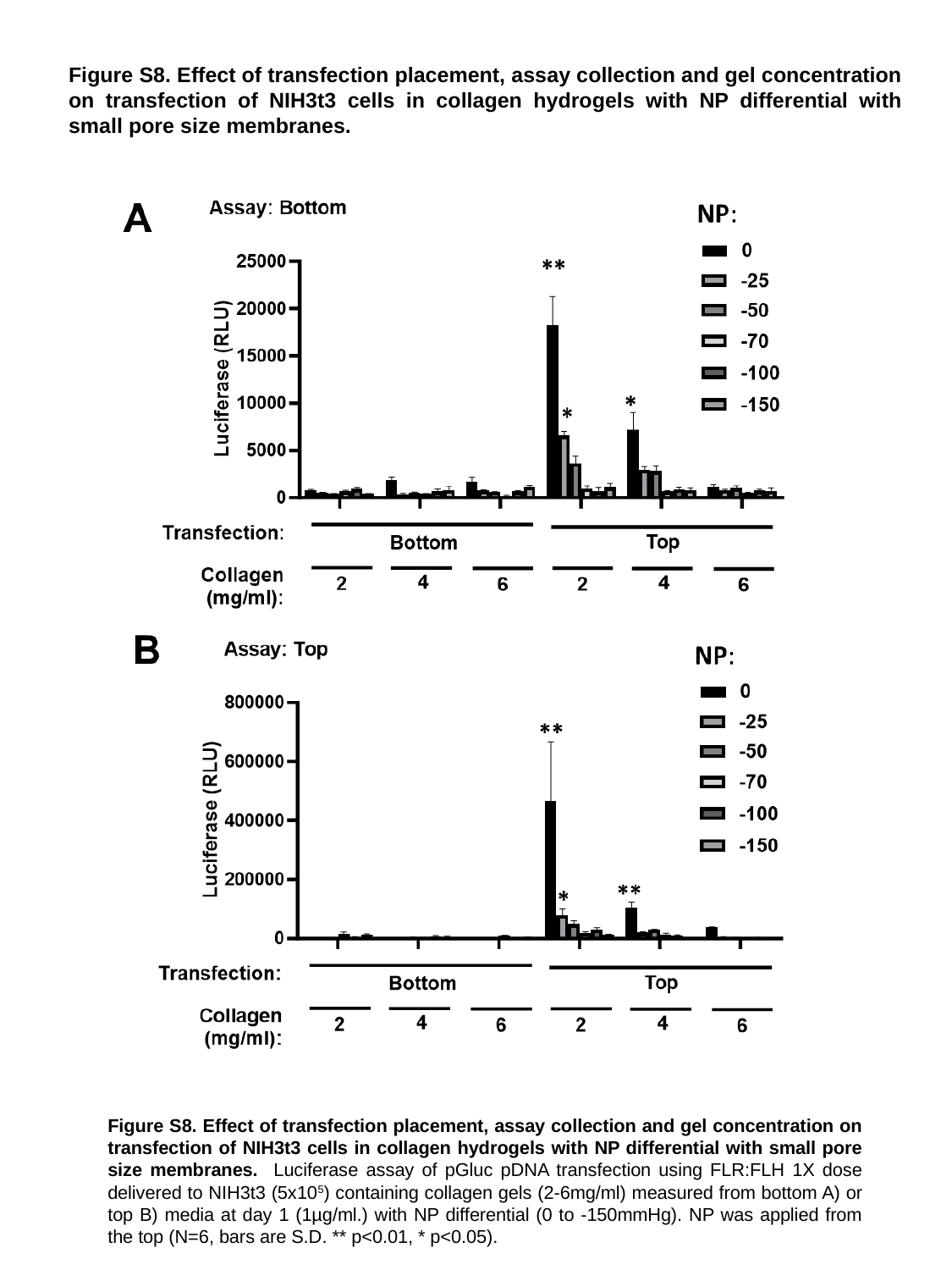

Figure S8. Effect of transfection placement, assay collection and gel concentration on transfection of NIH3t3 cells in collagen hydrogels with NP differential with small pore size membranes.
Figure S8. Effect of transfection placement, assay collection and gel concentration on transfection of NIH3t3 cells in collagen hydrogels with NP differential with small pore size membranes. Luciferase assay of pGluc pDNA transfection using FLR:FLH 1X dose delivered to NIH3t3 (5x10^5^) containing collagen gels (2-6mg/ml) measured from bottom A) or top B) media at day 1 (1µg/ml.) with NP differential (0 to -150mmHg). NP was applied from the top (N=6, bars are S.D. ** p<0.01, * p<0.05).

## Slide 9
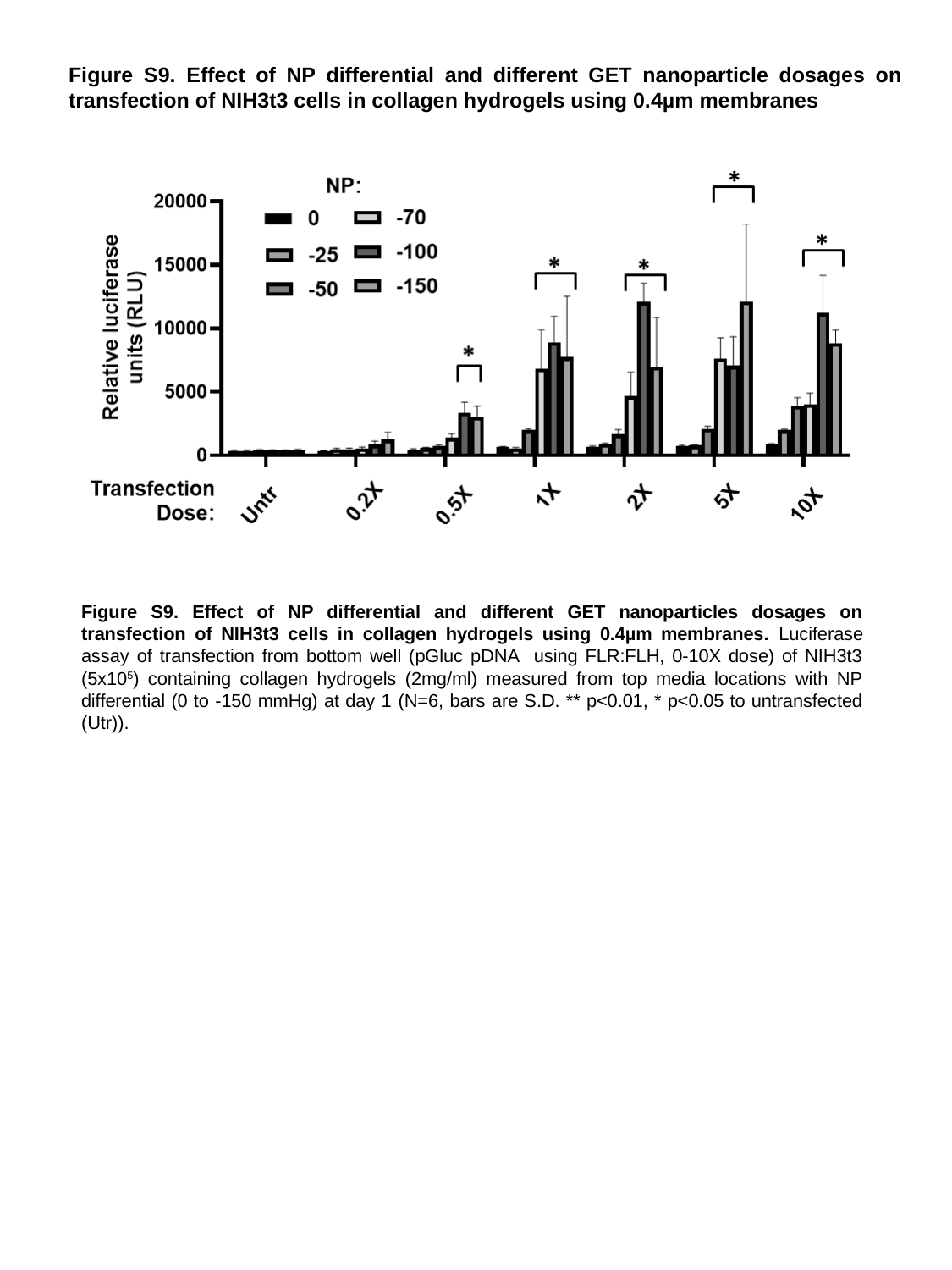

Figure S9. Effect of NP differential and different GET nanoparticle dosages on transfection of NIH3t3 cells in collagen hydrogels using 0.4µm membranes
Figure S9. Effect of NP differential and different GET nanoparticles dosages on transfection of NIH3t3 cells in collagen hydrogels using 0.4µm membranes. Luciferase assay of transfection from bottom well (pGluc pDNA using FLR:FLH, 0-10X dose) of NIH3t3 (5x10^5^) containing collagen hydrogels (2mg/ml) measured from top media locations with NP differential (0 to -150 mmHg) at day 1 (N=6, bars are S.D. ** p<0.01, * p<0.05 to untransfected (Utr)).

## Slide 10
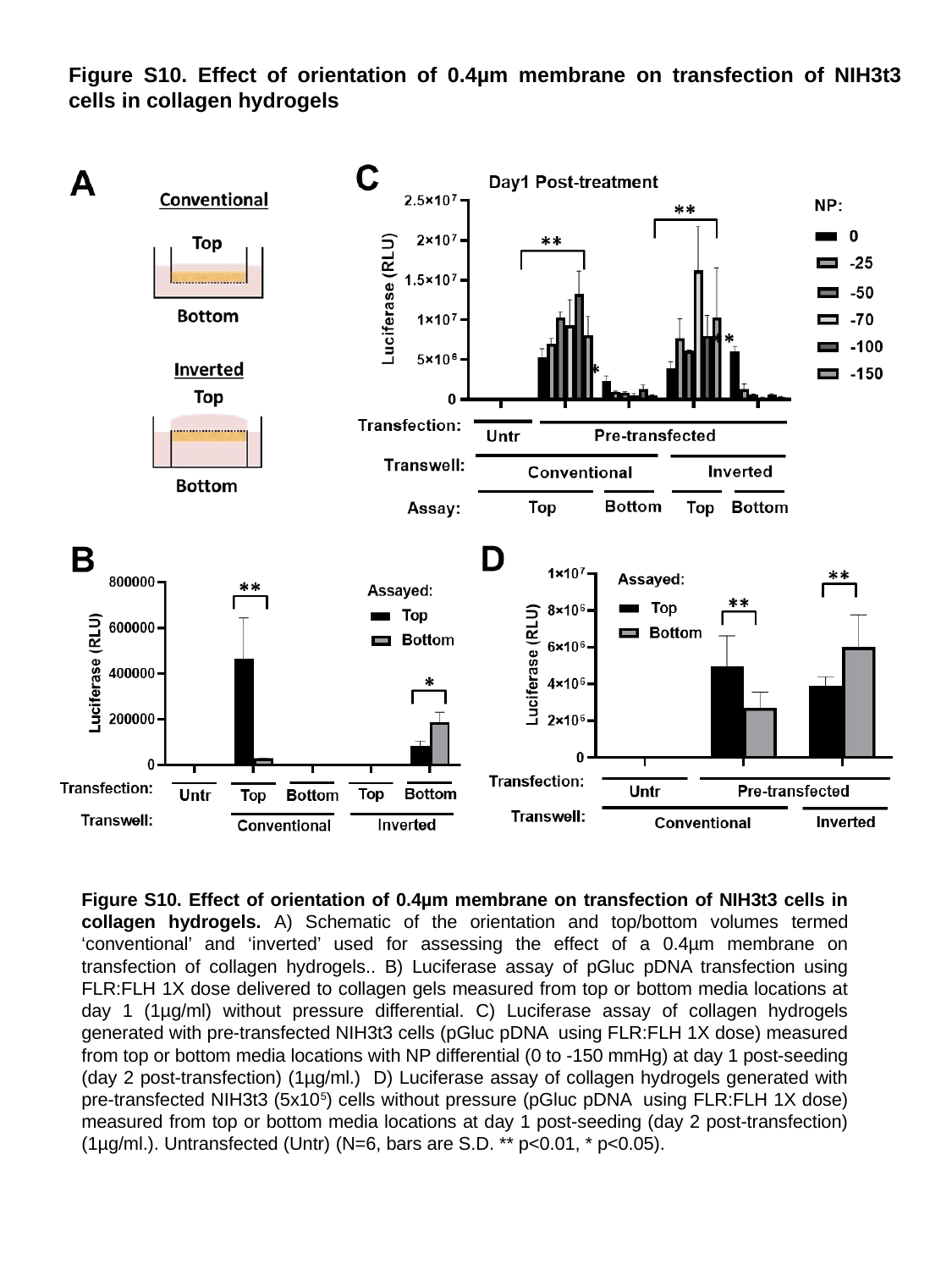

Figure S10. Effect of orientation of 0.4µm membrane on transfection of NIH3t3 cells in collagen hydrogels
Figure S10. Effect of orientation of 0.4µm membrane on transfection of NIH3t3 cells in collagen hydrogels. A) Schematic of the orientation and top/bottom volumes termed ‘conventional’ and ‘inverted’ used for assessing the effect of a 0.4µm membrane on transfection of collagen hydrogels.. B) Luciferase assay of pGluc pDNA transfection using FLR:FLH 1X dose delivered to collagen gels measured from top or bottom media locations at day 1 (1µg/ml) without pressure differential. C) Luciferase assay of collagen hydrogels generated with pre-transfected NIH3t3 cells (pGluc pDNA using FLR:FLH 1X dose) measured from top or bottom media locations with NP differential (0 to -150 mmHg) at day 1 post-seeding (day 2 post-transfection) (1µg/ml.) D) Luciferase assay of collagen hydrogels generated with pre-transfected NIH3t3 (5x10^5^) cells without pressure (pGluc pDNA using FLR:FLH 1X dose) measured from top or bottom media locations at day 1 post-seeding (day 2 post-transfection) (1µg/ml.). Untransfected (Untr) (N=6, bars are S.D. ** p<0.01, * p<0.05).

## Slide 11
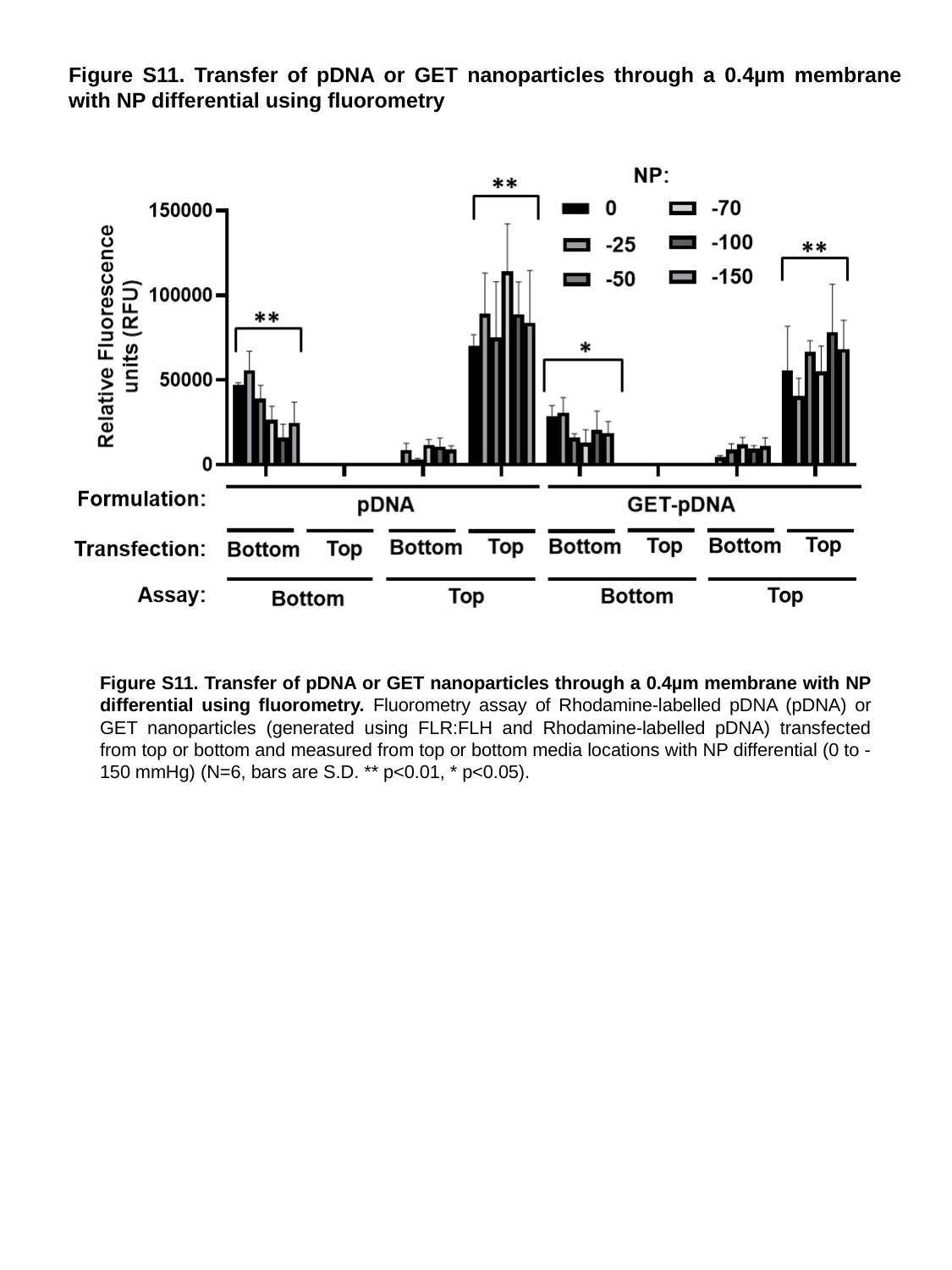

Figure S11. Transfer of pDNA or GET nanoparticles through a 0.4µm membrane with NP differential using fluorometry
Figure S11. Transfer of pDNA or GET nanoparticles through a 0.4µm membrane with NP differential using fluorometry. Fluorometry assay of Rhodamine-labelled pDNA (pDNA) or GET nanoparticles (generated using FLR:FLH and Rhodamine-labelled pDNA) transfected from top or bottom and measured from top or bottom media locations with NP differential (0 to -150 mmHg) (N=6, bars are S.D. ** p<0.01, * p<0.05).

## Slide 12
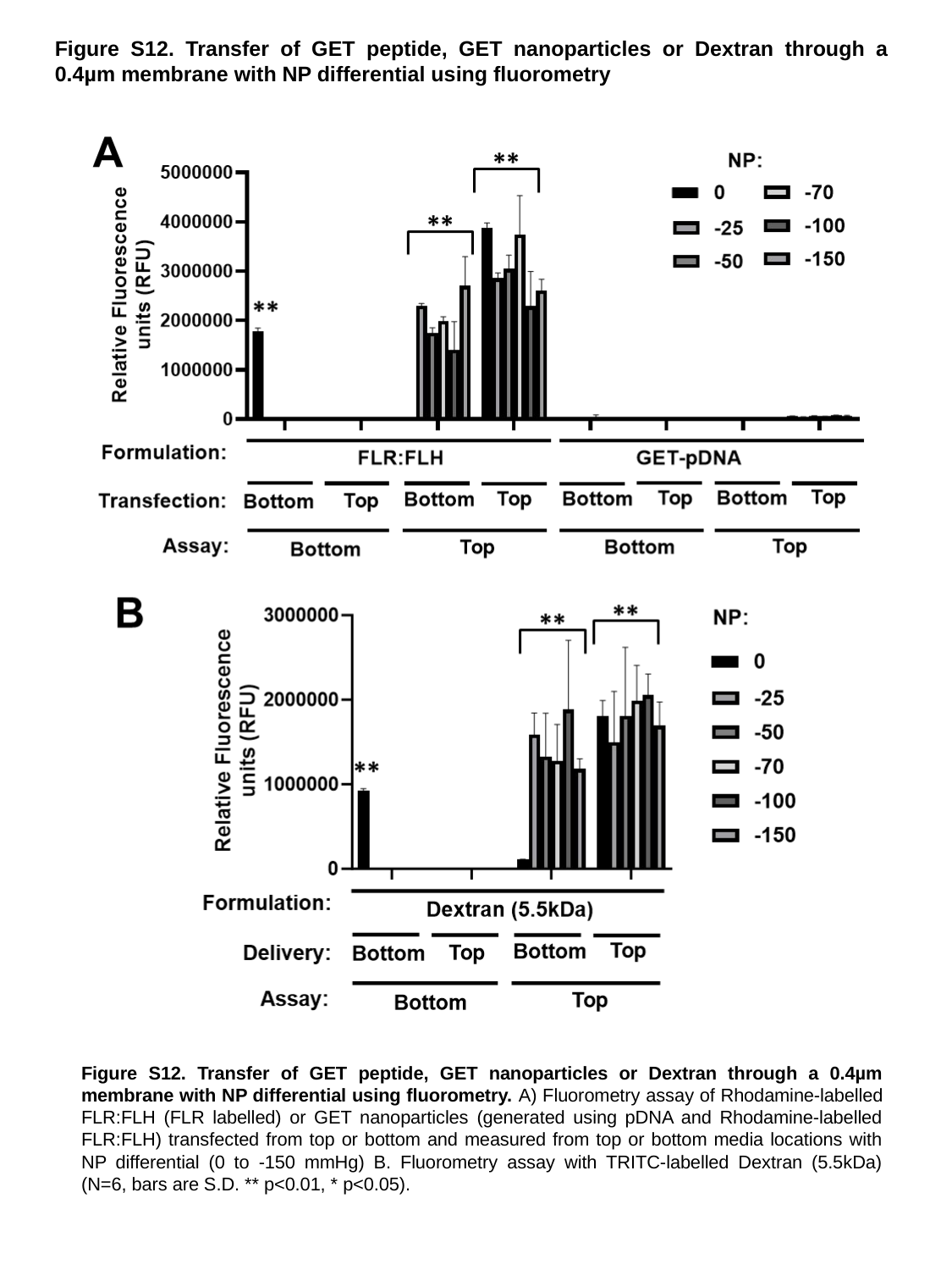

Figure S12. Transfer of GET peptide, GET nanoparticles or Dextran through a 0.4µm membrane with NP differential using fluorometry
Figure S12. Transfer of GET peptide, GET nanoparticles or Dextran through a 0.4µm membrane with NP differential using fluorometry. A) Fluorometry assay of Rhodamine-labelled FLR:FLH (FLR labelled) or GET nanoparticles (generated using pDNA and Rhodamine-labelled FLR:FLH) transfected from top or bottom and measured from top or bottom media locations with NP differential (0 to -150 mmHg) B. Fluorometry assay with TRITC-labelled Dextran (5.5kDa) (N=6, bars are S.D. ** p<0.01, * p<0.05).

## Slide 13
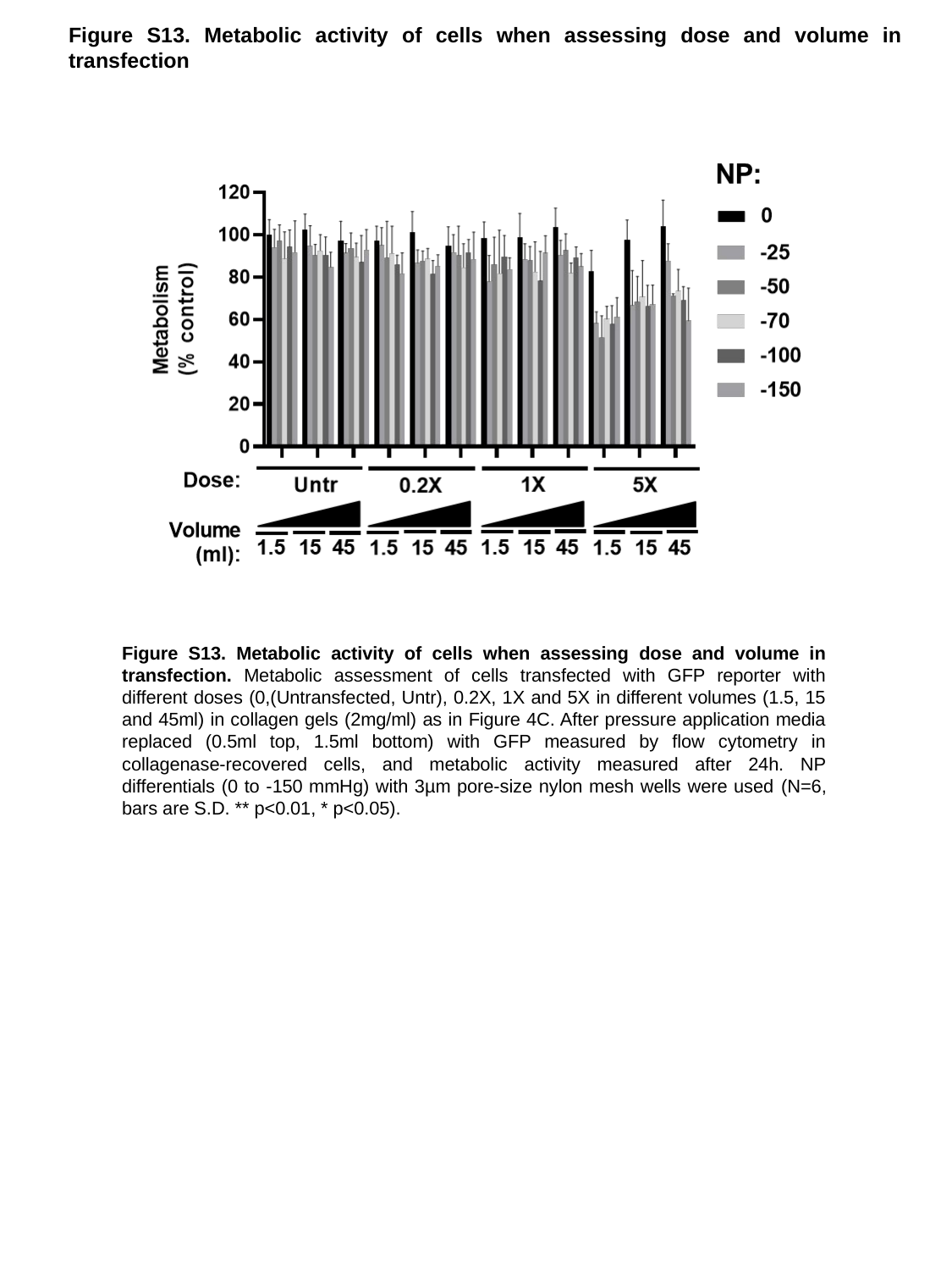

Figure S13. Metabolic activity of cells when assessing dose and volume in transfection
Figure S13. Metabolic activity of cells when assessing dose and volume in transfection. Metabolic assessment of cells transfected with GFP reporter with different doses (0,(Untransfected, Untr), 0.2X, 1X and 5X in different volumes (1.5, 15 and 45ml) in collagen gels (2mg/ml) as in Figure 4C. After pressure application media replaced (0.5ml top, 1.5ml bottom) with GFP measured by flow cytometry in collagenase-recovered cells, and metabolic activity measured after 24h. NP differentials (0 to -150 mmHg) with 3µm pore-size nylon mesh wells were used (N=6, bars are S.D. ** p<0.01, * p<0.05).

## Slide 14
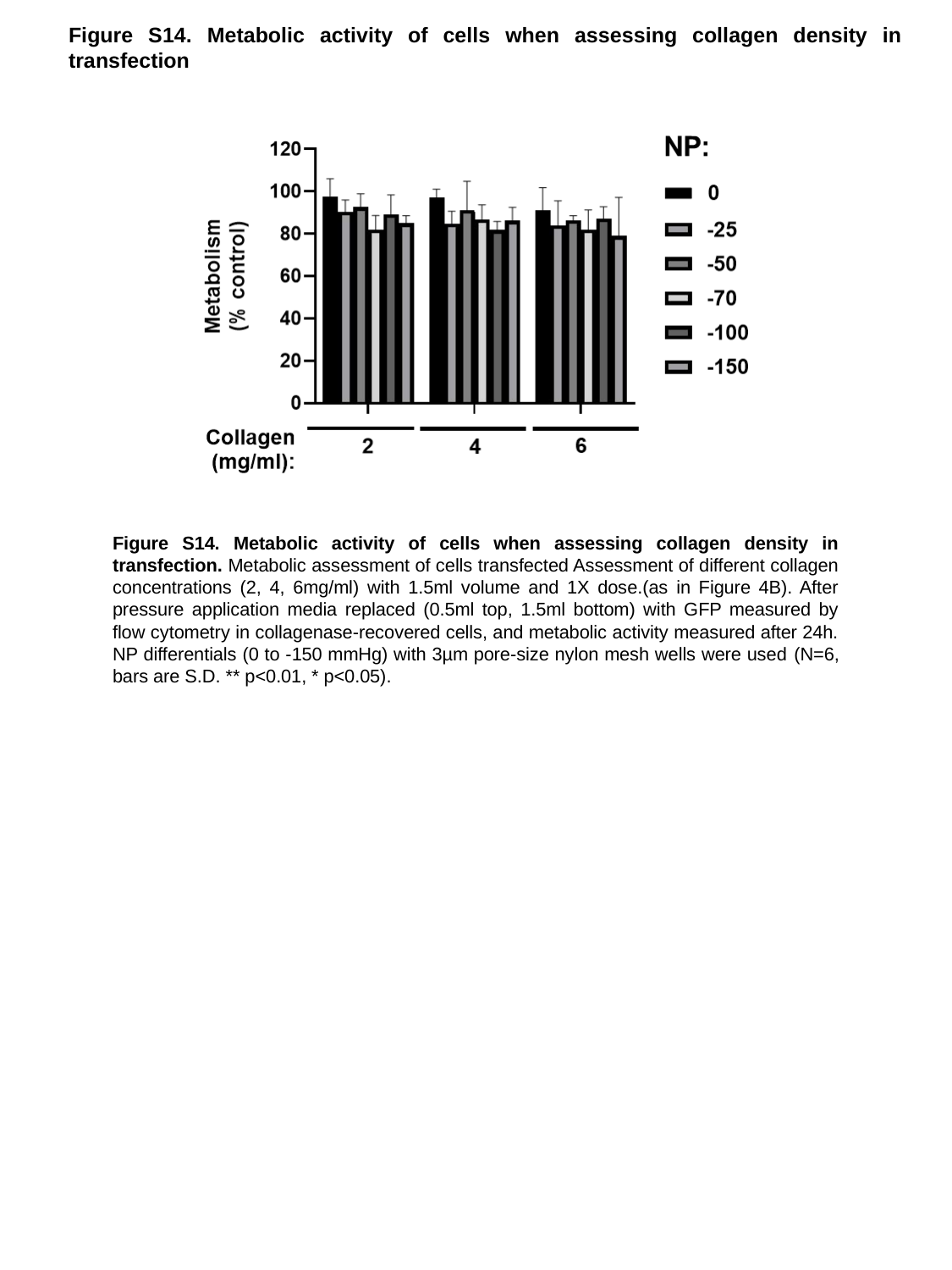

Figure S14. Metabolic activity of cells when assessing collagen density in transfection
Figure S14. Metabolic activity of cells when assessing collagen density in transfection. Metabolic assessment of cells transfected Assessment of different collagen concentrations (2, 4, 6mg/ml) with 1.5ml volume and 1X dose.(as in Figure 4B). After pressure application media replaced (0.5ml top, 1.5ml bottom) with GFP measured by flow cytometry in collagenase-recovered cells, and metabolic activity measured after 24h. NP differentials (0 to -150 mmHg) with 3µm pore-size nylon mesh wells were used (N=6, bars are S.D. ** p<0.01, * p<0.05).

## Slide 15
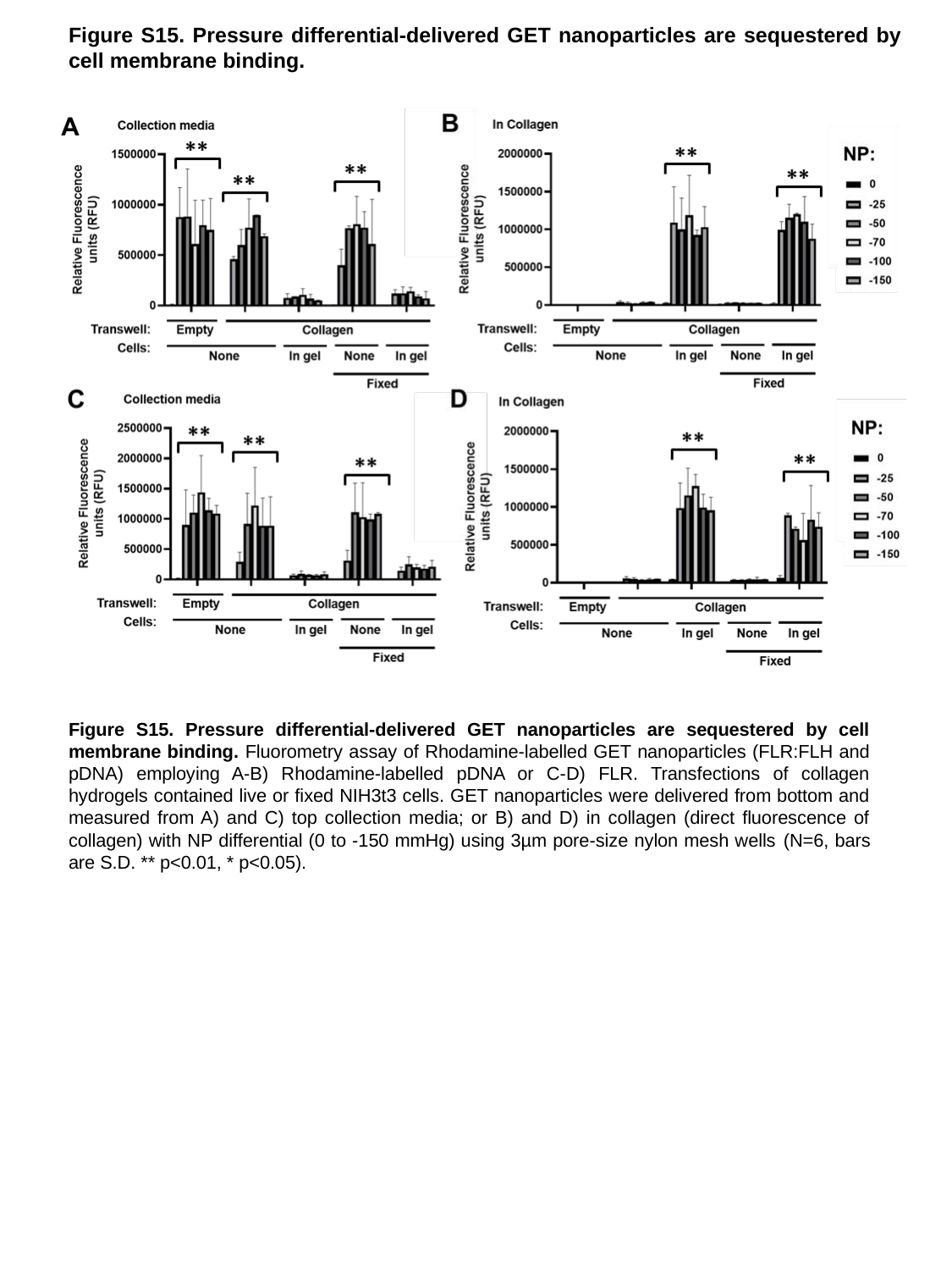

Figure S15. Pressure differential-delivered GET nanoparticles are sequestered by cell membrane binding.
Figure S15. Pressure differential-delivered GET nanoparticles are sequestered by cell membrane binding. Fluorometry assay of Rhodamine-labelled GET nanoparticles (FLR:FLH and pDNA) employing A-B) Rhodamine-labelled pDNA or C-D) FLR. Transfections of collagen hydrogels contained live or fixed NIH3t3 cells. GET nanoparticles were delivered from bottom and measured from A) and C) top collection media; or B) and D) in collagen (direct fluorescence of collagen) with NP differential (0 to -150 mmHg) using 3µm pore-size nylon mesh wells (N=6, bars are S.D. ** p<0.01, * p<0.05).

## Slide 16
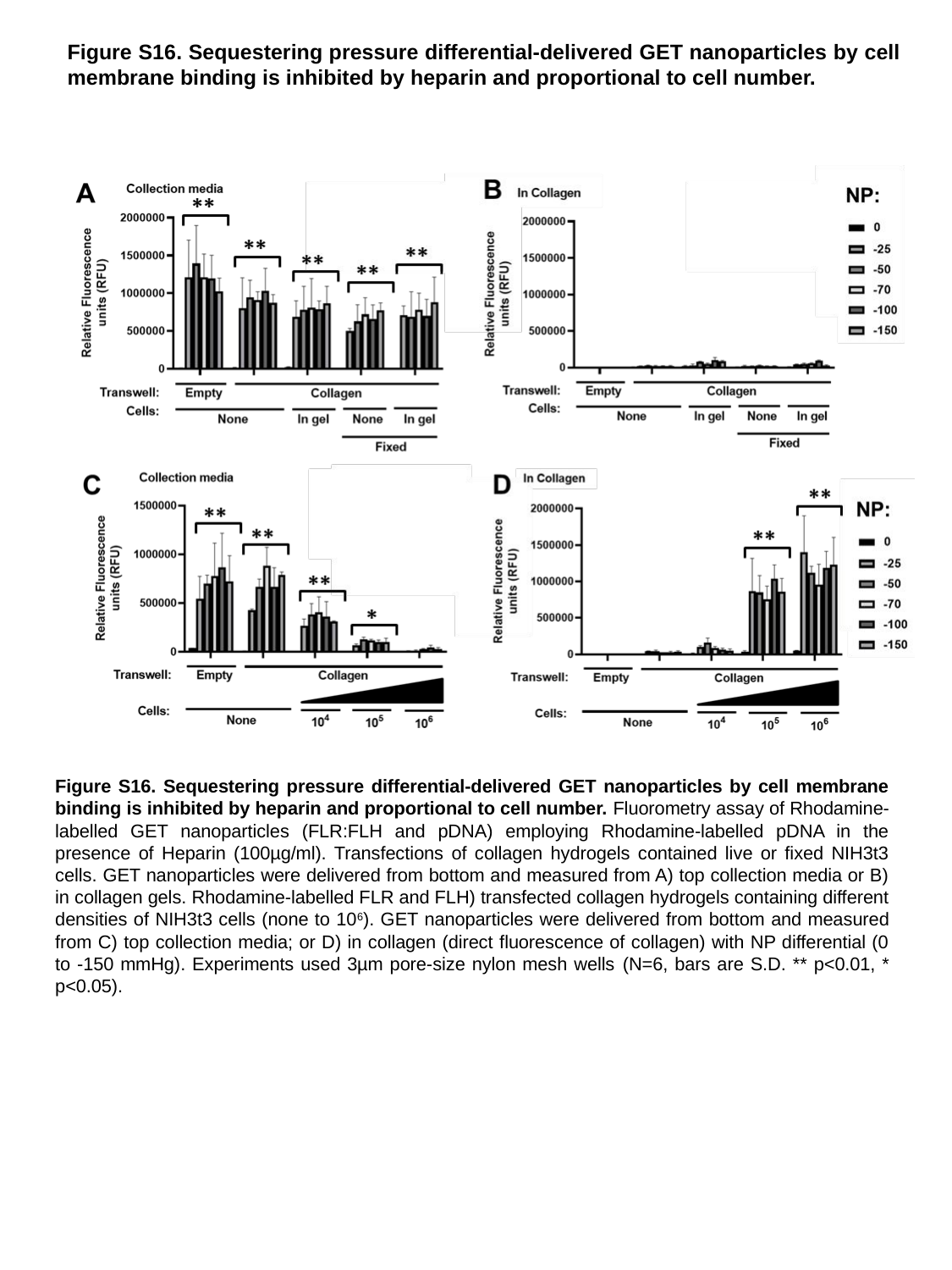

Figure S16. Sequestering pressure differential-delivered GET nanoparticles by cell membrane binding is inhibited by heparin and proportional to cell number.
Figure S16. Sequestering pressure differential-delivered GET nanoparticles by cell membrane binding is inhibited by heparin and proportional to cell number. Fluorometry assay of Rhodamine-labelled GET nanoparticles (FLR:FLH and pDNA) employing Rhodamine-labelled pDNA in the presence of Heparin (100µg/ml). Transfections of collagen hydrogels contained live or fixed NIH3t3 cells. GET nanoparticles were delivered from bottom and measured from A) top collection media or B) in collagen gels. Rhodamine-labelled FLR and FLH) transfected collagen hydrogels containing different densities of NIH3t3 cells (none to 106). GET nanoparticles were delivered from bottom and measured from C) top collection media; or D) in collagen (direct fluorescence of collagen) with NP differential (0 to -150 mmHg). Experiments used 3µm pore-size nylon mesh wells (N=6, bars are S.D. ** p<0.01, * p<0.05).

## Slide 17
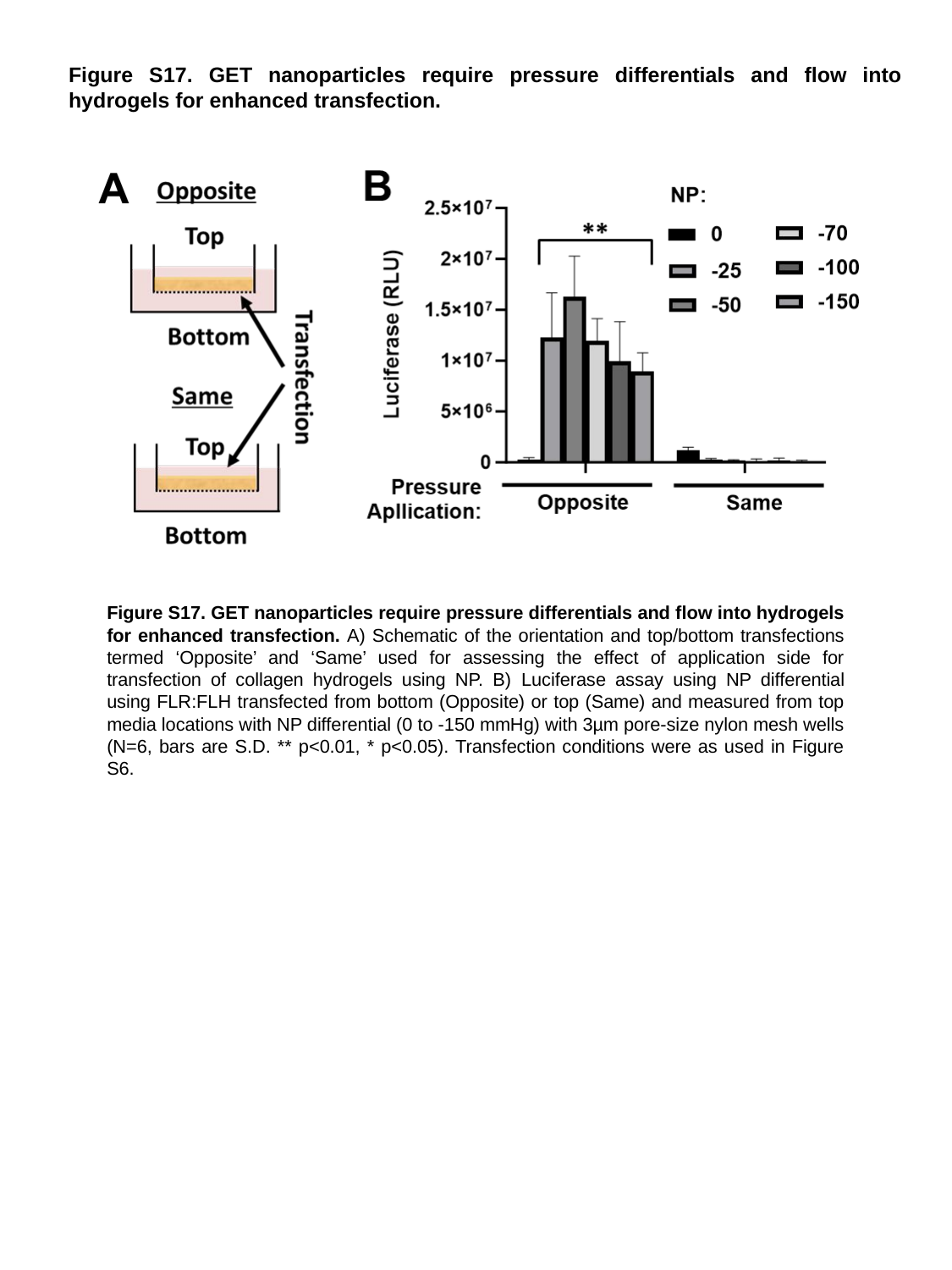

Figure S17. GET nanoparticles require pressure differentials and flow into hydrogels for enhanced transfection.
Figure S17. GET nanoparticles require pressure differentials and flow into hydrogels for enhanced transfection. A) Schematic of the orientation and top/bottom transfections termed ‘Opposite’ and ‘Same’ used for assessing the effect of application side for transfection of collagen hydrogels using NP. B) Luciferase assay using NP differential using FLR:FLH transfected from bottom (Opposite) or top (Same) and measured from top media locations with NP differential (0 to -150 mmHg) with 3µm pore-size nylon mesh wells (N=6, bars are S.D. ** p<0.01, * p<0.05). Transfection conditions were as used in Figure S6.

## Slide 18
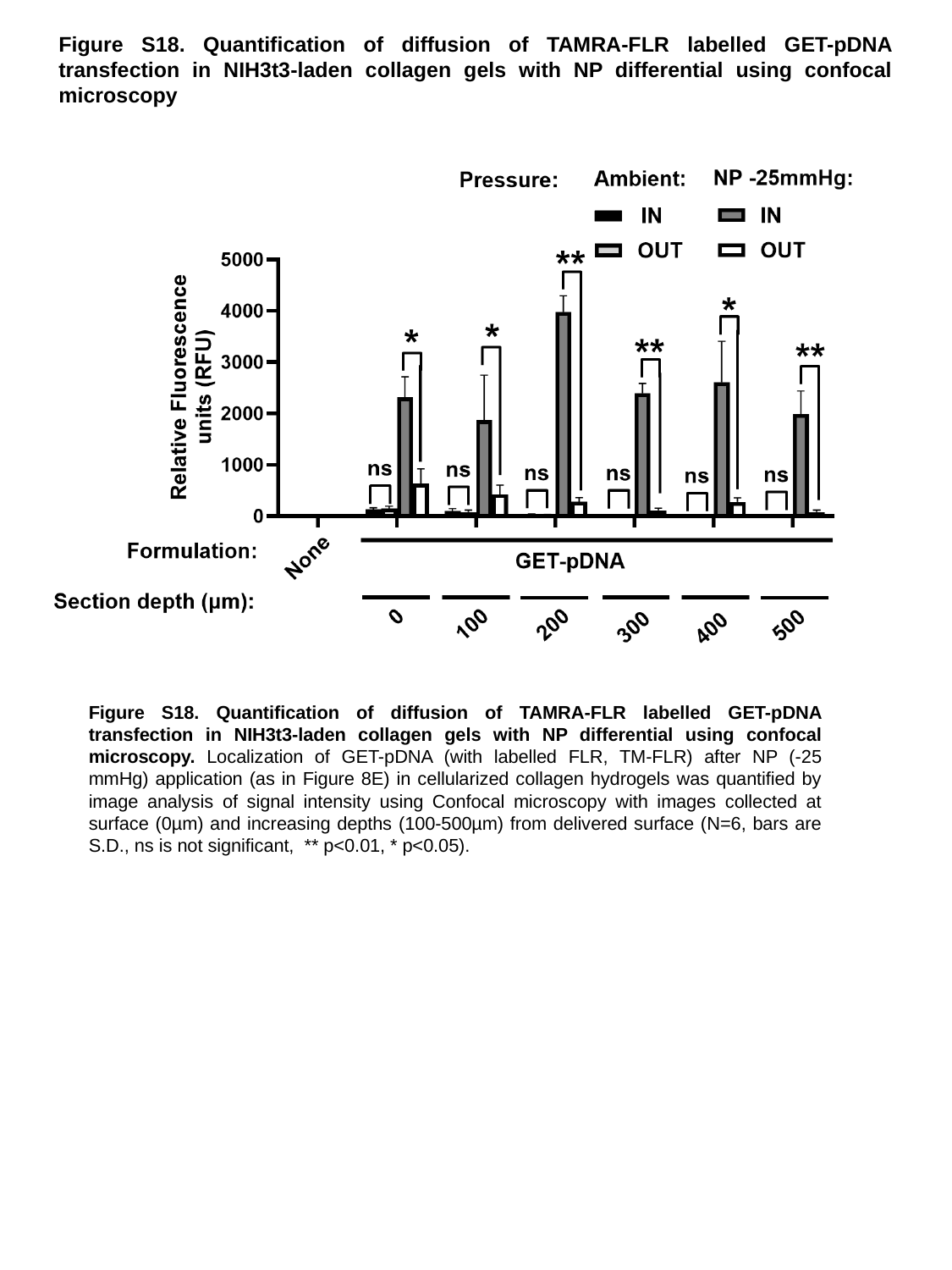

Figure S18. Quantification of diffusion of TAMRA-FLR labelled GET-pDNA transfection in NIH3t3-laden collagen gels with NP differential using confocal microscopy
Figure S18. Quantification of diffusion of TAMRA-FLR labelled GET-pDNA transfection in NIH3t3-laden collagen gels with NP differential using confocal microscopy. Localization of GET-pDNA (with labelled FLR, TM-FLR) after NP (-25 mmHg) application (as in Figure 8E) in cellularized collagen hydrogels was quantified by image analysis of signal intensity using Confocal microscopy with images collected at surface (0µm) and increasing depths (100-500µm) from delivered surface (N=6, bars are S.D., ns is not significant, ** p<0.01, * p<0.05).

## Slide 19
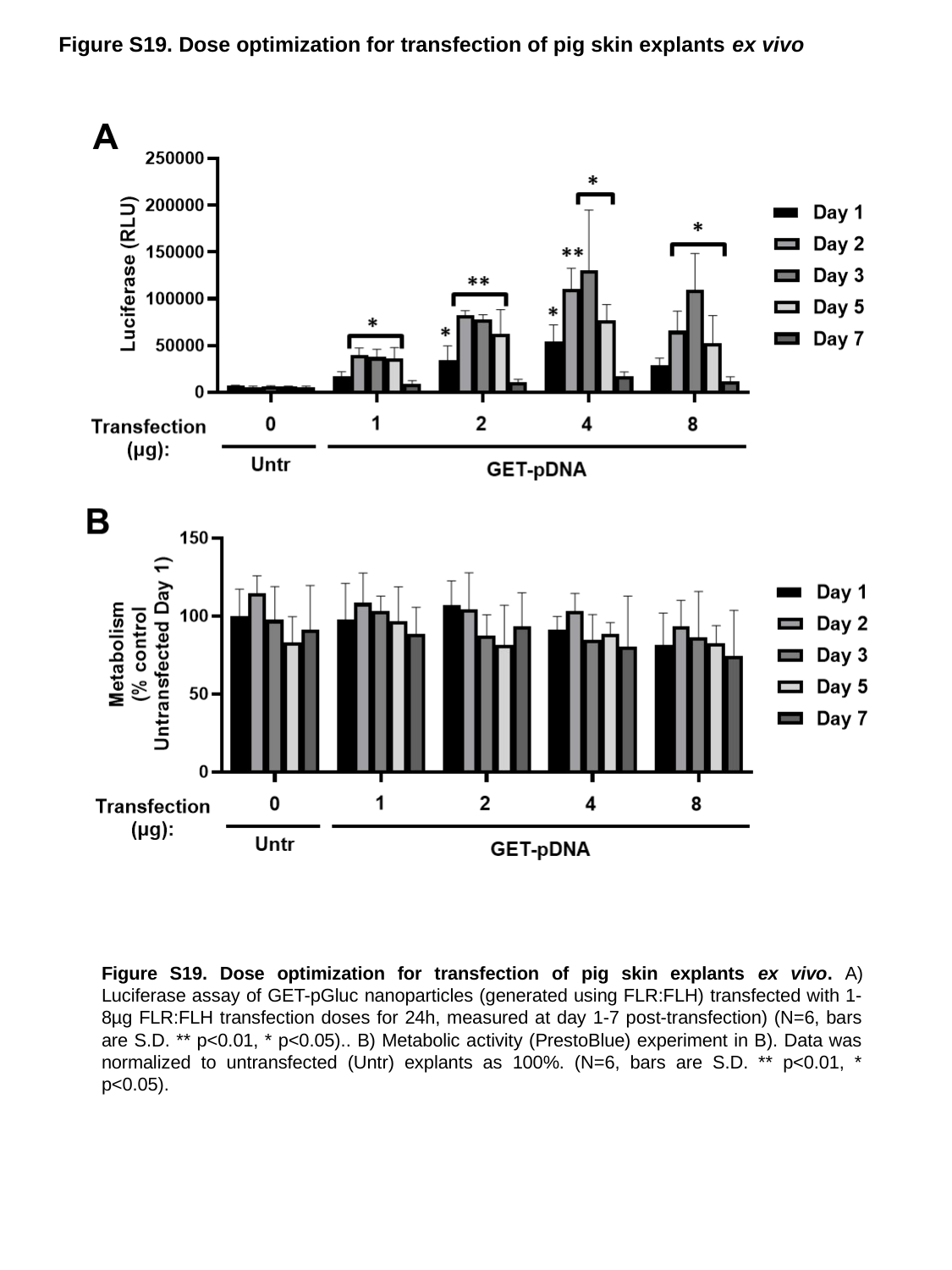

Figure S19. Dose optimization for transfection of pig skin explants ex vivo
Figure S19. Dose optimization for transfection of pig skin explants ex vivo. A) Luciferase assay of GET-pGluc nanoparticles (generated using FLR:FLH) transfected with 1-8µg FLR:FLH transfection doses for 24h, measured at day 1-7 post-transfection) (N=6, bars are S.D. ** p<0.01, * p<0.05).. B) Metabolic activity (PrestoBlue) experiment in B). Data was normalized to untransfected (Untr) explants as 100%. (N=6, bars are S.D. ** p<0.01, * p<0.05).
